# ArsRS-Dependent Regulation of *homB* Contributes to *Helicobacter pylori* Biofilm Formation

**DOI:** 10.3389/fmicb.2018.01497

**Published:** 2018-08-02

**Authors:** Stephanie L. Servetas, Ryan S. Doster, Aeryun Kim, Ian H. Windham, Jeong-Heon Cha, Jennifer A. Gaddy, D. Scott Merrell

**Affiliations:** ^1^Department of Microbiology and Immunology, Uniformed Services University of the Health Sciences, Bethesda, MD, United States; ^2^Department of Medicine, Vanderbilt University, Nashville, TN, United States; ^3^BK21 Plus Project, Department of Oral Biology, Oral Science Research Center, Yonsei University College of Dentistry, Seoul, South Korea; ^4^Department of Applied Life Science, The Graduate School, Yonsei University, Seoul, South Korea; ^5^Microbiology and Molecular Biology Laboratory, Key Laboratory of Oral Medicine, Guangzhou Institute of Oral Disease, Stomatology Hospital of Guangzhou Medical University, Guangzhou, China; ^6^Tennessee Valley Healthcare Systems, Department of Veterans Affairs, Nashville, TN, United States

**Keywords:** *homB*, ArsRS, *H. pylori*, biofilms, OMPs

## Abstract

One elusive area in the *Helicobacter pylori* field is an understanding of why some infections result in gastric cancer, yet others persist asymptomatically for the life-span of the individual. Even before the genomic era, the high level of intraspecies diversity of *H. pylori* was well recognized and became an intriguing area of investigation with respect to disease progression. Of interest in this regard is the unique repertoire of over 60 outer membrane proteins (OMPs), several of which have been associated with disease outcome. Of these OMPs, the association between HomB and disease outcome varies based on the population being studied. While the molecular roles for some of the disease-associated OMPs have been evaluated, little is known about the role that HomB plays in the *H. pylori* lifecycle. Thus, herein we investigated *homB* expression, regulation, and contribution to biofilm formation. We found that in *H. pylori* strain G27, *homB* was expressed at a relatively low level until stationary phase. Furthermore, *homB* expression was suppressed at low pH in an ArsRS-dependent manner; mutation of *arsRS* resulted in increased *homB* transcript at all tested time-points. ArsRS regulation of *homB* appeared to be direct as purified ArsR was able to specifically bind to the *homB* promoter. This regulation, combined with our previous finding that ArsRS mutations lead to enhanced biofilm formation, led us to test the hypothesis that *homB* contributes to biofilm formation by *H. pylori*. Indeed, subsequent biofilm analysis using a crystal-violet quantification assay and scanning electron microscopy (SEM) revealed that loss of *homB* from hyper-biofilm forming strains resulted in reversion to a biofilm phenotype that mimicked wild-type. Furthermore, expression of *homB in trans* from a promoter that negated ArsRS regulation led to enhanced biofilm formation even in strains in which the chromosomal copy of *homB* had been deleted. Thus, *homB* is necessary for hyper-biofilm formation of ArsRS mutant strains and aberrant regulation of this gene is sufficient to induce a hyper-biofilm phenotype. In summary, these data suggest that the ArsRS-dependent regulation of OMPs such as HomB may be one mechanism by which ArsRS dictates biofilm development in a pH responsive manner.

## Introduction

*Helicobacter pylori* is an intriguing pathogen. Not only does it colonize the inhospitable gastric niche, but it does so with incredible efficiency. It is estimated that approximately 50% of the world's population is colonized by *H. pylori* (Goh et al., 2011). In a majority of cases, infection with *H. pylori* remains in a relatively quiescent, asymptomatic state. For those individuals that develop *H. pylori*-associated diseases, symptomatic *H. pylori* infections can present as gastritis, duodenal or gastric ulcers, gastric adenocarcinoma, or mucosa associated lymphoid tissue (MALT) lymphoma (Bauer and Meyer, 2011). Given the broad range of clinical manifestations associated with *H. pylori* infection, one of the most tantalizing avenues of research is identification of the bacterial factors that dictate disease progression. Indeed, the extensive heterogeneity of the *H. pylori* genome makes this pathogen an attractive target in the hunt for factors that can predict disease outcome.

Greater than 20% of *H. pylori'*s gene content varies between strains (Dong et al., 2009). Many of these auxiliary genes encode for proteins of unknown function; however, genes of known function include those encoding for DNA modifying proteins, LPS modifying proteins, outer membrane proteins, and members of the Cag pathogenicity island (PAI) (Dong et al., 2009). In fact, one of the main predictors of severe disease outcome, particularly gastric cancer, is the presence of the cytotoxin, CagA, which is encoded on the PAI. Interestingly, while CagA-positive strains are associated with an increased risk for development of gastric cancer as compared to CagA-negative strains, the risk among CagA-positive strains is further delineated based on conserved polymorphisms in the CagA C-terminal EPIYA motif (Cover, 2016). Thus, as demonstrated by the previous example, *H. pylori*'s panmictic structure is based not only on the presence or absence of genes, but also on conserved polymorphisms within certain genes.

Another class of genes that has been of particular interest in terms of an association with disease outcomes are those that encode for outer membrane proteins (OMPs) (Cover, 2016). Gram-negative OMPs are unique because, unlike many integral membrane proteins that are comprised of alpha helices, OMPs are beta-barrel proteins (Koebnik et al., 2000; Schulz, 2002; Wimley, 2003). OMPs have been shown to function in a variety of roles: molecular transportation, antibiotic resistance, and virulence (Koebnik et al., 2000; Schulz, 2002; Wimley, 2003). For a pathogen that resides within a very specific niche, *H. pylori* encodes an extensive array of OMPs. In the majority of Gram-negative bacteria, two to three percent of the genome is typically dedicated to OMPs (Wimley, 2003). For a bacterium such as *Haemophilus influenza*, which has a genome size similar to *H. pylori*, this equates to approximately 40 proteins (Wimley, 2003). In comparison, up to 60 OMPs are predicted to be encoded by *H. pylori*'s relatively small genome (1.6 Mb); this is equal to approximately 4% of the genome (Alm and Trust, 1999; Alm et al., 2000). This number becomes even more impressive when one considers that *H. pylori* is only predicted to encode 17 regulatory proteins (Scarlato et al., 2001).

Several *H. pylori* OMPs have been identified as adherence factors (reviewed in Oleastro and Menard, 2013). While adhesins may not directly damage the host, adherence is likely paramount to virulence for a persistent pathogen like *H. pylori*. This is supported by the fact that CagA is delivered to cells in a contact dependent manner. Without an adherent population, CagA would not reach the host epithelium (Jones et al., 2010). Indeed, in some strains the presence of *hopQ* was found to be required for CagA translocation (Belogolova et al., 2013). Additionally, the intimate attachment of *H. pylori* to the gastric epithelium is well documented (Schreiber et al., 2004) and believed to be crucial for maintenance of long term infection (Blaser and Kirschner, 1999). Finally, given that surface adherence is the initiating step in biofilm formation, OMPs likely contribute to biofilm formation as well (O'Toole et al., 2000). In fact, biofilm formation is thought to contribute to many chronic bacterial infections, and the presence of *H. pylori* biofilms *in vivo* has been observed (Carron et al., 2006; Coticchia et al., 2006; Cellini et al., 2008; Cammarota et al., 2010).

The high level of genetic diversity seen with the OMPs has led several groups to investigate the association between specific OMP(s) and clinical outcomes (reviewed in Oleastro and Menard, 2013). One OMP that shows an association with more severe disease outcomes is HomB. *homB* was identified in a screen for genes associated with formation of duodenal ulcers; the authors compared isolates from a duodenal ulcer patient and a gastritis patient (Oleastro et al., 2006). Further analysis showed that in individuals less than 40 years of age, *homB* was significantly associated with peptic ulcer disease (Oleastro et al., 2006, 2008, 2009b). Following this initial publication, several groups have studied the association between *homB* and disease severity in a variety of populations (Oleastro et al., 2008, 2009b; Jung et al., 2009; Talebi Bezmin Abadi et al., 2011; Kang et al., 2012).

Despite these initial findings, in comparison to other OMPs that are associated with disease outcomes, very little is known about this OMP or the larger Hom family at a molecular level. From the original genome publication, we know that HomB is a member of a small OMP family, the *Helicobacter* outer membrane protein or Hom family. There are four members that make up the *hom* family: HomA, HomB, HomC, and HomD. Based on the few molecular studies that have been conducted, HomB appears to be involved in adherence (Oleastro et al., 2008; Oleastro and Menard, 2013). Furthermore, serological evidence suggests that HomB is expressed *in vivo* (Oleastro et al., 2008). Additionally, two interesting observations that arose from the epidemiological studies were that (1) *homB* could occupy two different loci, locus A and locus B, and (2) strains can carry a single or double copy of *homB* (Oleastro et al., 2009a, 2010; Kang et al., 2012). To date, neither phase variation nor recombination has been demonstrated for *homB*; however, differences in distribution of *homB* location and copy number have been reported (Oleastro et al., 2009a; Servetas et al., 2018). This is particularly intriguing given that the *homB* genotype varies between Western and East Asian isolates and because the association between *homB* and disease severity is dependent on the population being studied (Oleastro et al., 2008, 2009b; Jung et al., 2009; Hussein, 2011; Talebi Bezmin Abadi et al., 2011; Kang et al., 2012).

Given the lack of molecular detail concerning HomB, we compared the promoter regions of locus A and locus B, assessed *homB* expression, and examined the role of HomB in *H. pylori* biofilm formation. Our data indicate that both *homB* loci have nearly identical promoter regions, which we reason may maintain the presence of transcriptional binding sequences. Interestingly, subsequent evaluation of *homB* expression revealed that *homB* was suppressed in response to low pH and that this regulation occurs via the ArsRS two component system. ArsRS is an acid responsive two component system that is comprised of a sensor kinase, ArsS, and a response regulator, ArsR. ArsS autophosphorlyates in response to a decrease in pH, and then transfers the phosphate to ArsR, which then acts to repress or activate expression of downstream genes. Previous work from our lab identified an enhanced biofilm phenotype in strains in which ArsRS was mutated (Servetas et al., 2016). Herein, we demonstrate that *homB* contributes to ArsRS-induced biofilm formation by *H. pylori*.

## Materials and methods

### Bacterial strains and growth conditions

Strains and plasmids used in this study are listed in Table 1. Unless noted otherwise, *H. pylori* strains were grown as previously described (Carpenter et al., 2015). Briefly, *H. pylori* stock cultures were maintained at −80°C in *H. pylori* freezing media [brain heart infusion broth (BD Biosciences) containing 10% fetal bovine serum (FBS) and 20% glycerol (EMD chemicals, Inc)]. Freezer stocks were plated on horse blood agar (HBA) plates comprised of 4% Columbia agar (Neogean Corporation), 5% defibrinated horse blood (HemoStat Laboratoris, Dixon, CA), 2 mg/mL β-cyclodextran (Sigma), and an antibiotic/antifungal cocktail [10 μg/ml vancomycin (Amresco), 5 μg/ml cefsulodin (Sigma), 2.5 U/ml polymyxin B (Sigma), 5 μg/ml trimethoprim (Sigma), and 8 μg/ml amphotericin B (Amresco)]. Following growth on HBA plates, *H. pylori* strains were grown in liquid culture. Liquid media consisted of brucella broth (Neogen Corporation) supplemented with 10% FBS (Gibco) and 10 ug/mL vancomycin. Where indicated, 25 μg/mL Kanamycin (Kan^25^) was used to supplement the media. All cultures were grown at 37°C, in gas evacuation jars, under microaerobic conditions (5% O_2_, 10% CO_2_, and 85% N_2_) generated with an Anoxomat gas evacuation and replacement system (Advanced Instruments, Inc.); in addition, liquid cultures were grown shaking at 100 rpm.

**Table 1:** Plasmids and strains used in this study.

	**Description**	**References**
**PLASMIDS**		
pDSM 3	pKSF-II	Copass et al., 1997
pDSM 215	pTM117 promoterless vector	Carpenter et al., 2007
pDSM 463	pTM117::G27-HP0073 promoter region	Carpenter et al., 2015
pDSM 920	pBluescript::Δ*arsS*::kan	Wen et al., 2006
pDSM 1530	pGEM-T Easy::HPG27_667::*kansacB*	This study
pDSM 1670	pGEM-T Easy::G27Δ*hom*	This study
pDSM 1532	pTM117::pUreA*-homB*	This study
pDSM 1538	pTM117::pFlaA*-homB*	This study
**STRAINS**		
DSM 1	WT G27	Baltrus et al., 2009
DSM 215	G27 pTM117, Kan^R^	Carpenter et al., 2007
DSM 983	G27Δ*arsS* markerless	Carpenter et al., 2015
DSM 1442	G27Δ*hom* markerless	This study
DSM 1443	G27 Δ*arsSΔhom*	This study
DSM 1446	G27 ArsRD52N, unphosphorylatable, Kan^R^	Servetas et al., 2016
DSM 1530	*E.coli top10* pGEM HPG27_667*::kansacB*	This study
DSM 1531	*G27 HPG27_667::kansacB*	This study
DSM 1532	Top10 pUreA-*homB*	This study
DSM 1533	G27 pUreA-*homB*	This study
DSM 1538	Top10 pFlaA-*homB*	This study
DSM 1539	G27 pFlaA-*homB*	This study
DSM 1540	G27Δ*hom* pFlaA-*homB*	This study
DSM 1543	*H. pylori* G27 ArsR-D52N Δ*hom*	This study
DSM 1547	*H. pylori* G27Δ*hom* pTM117	This study
DSM 1546	G27Δ*hom* pUreA-*homB*	This study
**STRAINS FOR LOCUS A AND LOCUS B PROMOTER ANALYSIS**		
DSM 48	*H. pylori* strain 7.13	Franco et al., 2005
K42	Korean clinical isolate	Kang et al., 2012
K248	Korean clinical isolate	Kang et al., 2012
K23	Korean clinical isolate	Kang et al., 2012
K16	Korean clinical isolate	Kang et al., 2012
K82	Korean clinical isolate	Kang et al., 2012
K34	Korean clinical isolate	Kang et al., 2012
K183	Korean clinical isolate	Kang et al., 2012
K25	Korean clinical isolate	Kang et al., 2012
K209	Korean clinical isolate	Kang et al., 2012
K36	Korean clinical isolate	Kang et al., 2012
K44	Korean clinical isolate	Kang et al., 2012
K131	Korean clinical isolate	Kang et al., 2012
K3	Korean clinical isolate	Kang et al., 2012
K197	Korean clinical isolate	Kang et al., 2012
K10	Korean clinical isolate	Kang et al., 2012
K165	Korean clinical isolate	Kang et al., 2012
K26	Korean clinical isolate	Kang et al., 2012
K259	Korean clinical isolate	Kang et al., 2012
K104	Korean clinical isolate	Kang et al., 2012
K107	Korean clinical isolate	Kang et al., 2012
K197	Korean clinical isolate	Kang et al., 2012

### Sequencing of *homB* promoter regions

**Figure 1:**
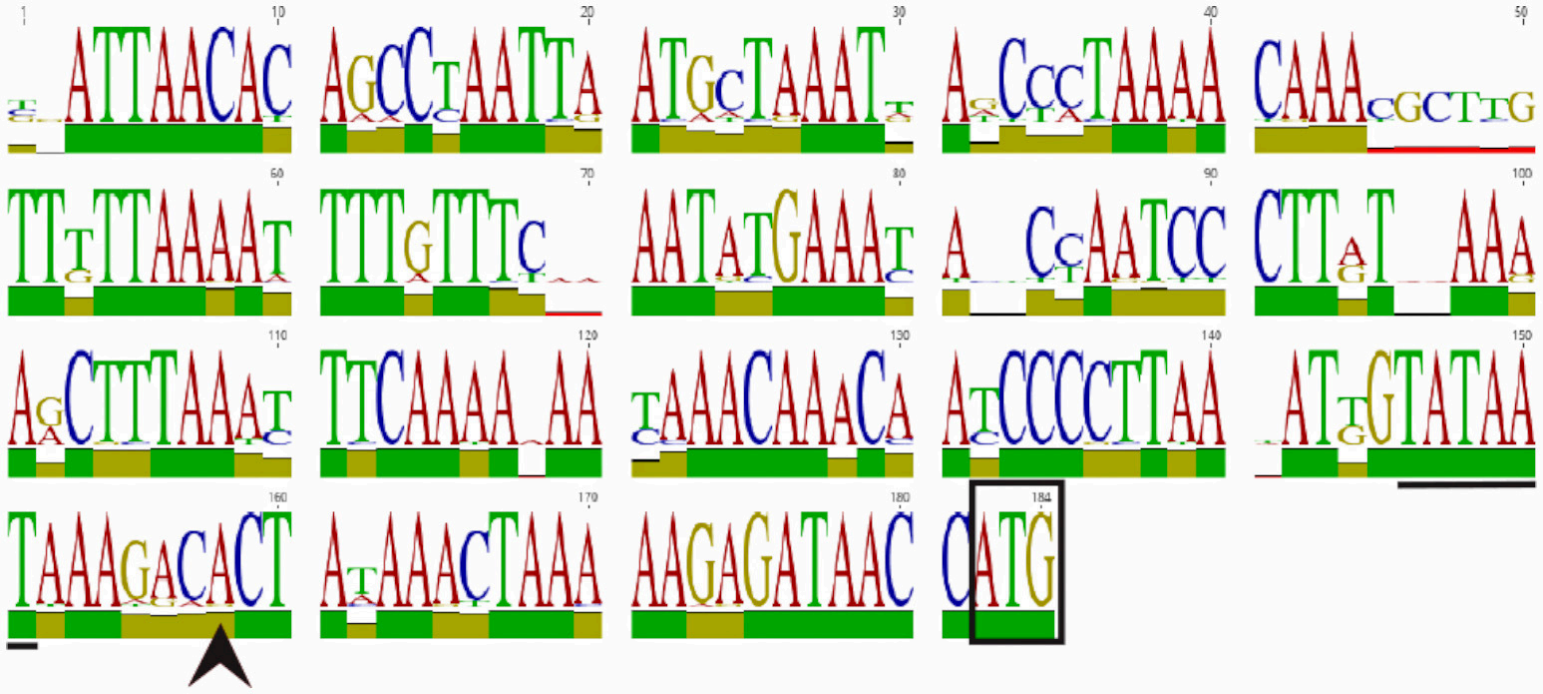
WebLogo of the *homB* promoter region. An alignment and subsequent WebLogo were generated from sequences from 30 *homB* loci from 29 strains. The overall conservation of the 184 nucleotides upstream of the *homB* genes from locus A and locus B are shown. The boxed ATG is the start codon for the *homB* gene. The +1 transcriptional start site (black arrowhead) and the−10 sequence (underlined) are both indicated. The larger the letter, the more highly conserved the corresponding base pair. To indicate nucleotides where substitutions/deletions are present, the letter(s) at these positions do not reach the full height. The overall conservation of each nucleotide position is indicated by the colored bar: green = 100%, tan = 30–99%, red < 30%.

Twenty-one *homB* positive strains from a collection of South Korean *H. pylori* clinical isolates (K42, K248, K23, K16, K82, K34, K183, K25, K209, K36, K44, K131, K3, K197, K10, K165, K26, K259, K104, K107, K197) were chosen for promoter analysis (Table 1; Kang et al., 2012). *hom* locus A and locus B were amplified from these 21 clinical isolates and from DSM48 using the AF/AR and BF/BR primer pairs, respectively (Table 2). Sanger sequencing of the promoter regions was carried out with the forward primers (Af and Bf) of each locus; these promoter sequences were deposited in the NCBI GenBank database with the accession numbers MF572264 to MF572284. The transcriptional start site was identified using the predictive data from Sharma et al. (2010). The *homB* promoter regions from a handful of *H. pylori* genomes that are available in Pubmed (SJM180, J99, HUP-B14, Gambia94/24, G27, P12, HPAG1) were also used to supplement the data from the clinical isolates. In total, 30 sequences from 29 strains (both locus A and locus B from strain DSM48 were included in the analysis) that extend 180 bp upstream from the *homB* start codon were compiled and aligned using the Geneious software 9.1.6 (Biomatters Ltd.) (Supplementary Figure 1). A Weblogo was created based on nucleotide conservation (Figure 1). In addition, the *homB* promoter region from G27 was also run through the Basic Local Alignment Search Tool (BLAST) to get a more global view of the sequence conservation among *homB* promoters.

**Table 2:** Primers used in this study.

**Primer name**	**Sequence (5′−3′)**	**References**
**Δ*****hom*** **CONSTRUCTION PRIMERS^A^**		
K4A	GTGCTCGAGCCCGGGCGAACCATTTGAGGTGA	Copass et al., 1997
PFA3	GCTCTAGACCCGGGTATAAGCCCATTTTCATGC	Copass et al., 1997
LocA_UpF	GTAAATCGGTGGTATTGAAG	This study
LocA_UpR	TCACCTCAAATGGTTCGCCCGGGCTCGAGCACATATCAATATTCCAGTCAGCAC	This study
LocA_DnF	GCATGAAAATGGGCTTATACCCGGGTCTAGAGCATCAACGCTTCTTAATAAGC	This study
LocA_DnR	ACAAATGATCGTTATTGTGG	This study
**ArsR D52N CONFIRMATION PRIMERS**		
Up1F	GGGGATTTTTTGAGCGTTGAG	Servetas et al., 2016
Dn4R	CGCAAACGGCCAATGATCAC	Servetas et al., 2016
**Δ*****arsS*** **CONFIRMATION PRIMERS**		
HP0165_del_ver_F	TGAAAGCATTGCGATTGAGA	Servetas et al., 2016
HP0165_del_ver_R	AAAACGGCTTTGATGCCTAA	Servetas et al., 2016
***hom*** **EXPRESSION VECTOR CONTRUCTION PRIMERS^B^**		
G27homB_FXbaI	agagag*TCTAGA*ATGAGAAAACTATTCATCCCACTTTTATTATTC	This study
G27homB_RPstI	agagag*CTGCAG*CACGCTCAAAACACCCAC	This study
G27pflaA_FKpnI	agagag*GGTACC*AGCCCATTTTCATGCTCCTAATT	This study
G27pflaA_RXbaI	agagag*TCTAGA*TTATAAAAAACCCAAAGGCATCCTTG	This study
**PROMOTER SEQUENCING PRIMERS**		
Af (F1-jhp0648/HP0709)	TAATTTCGCGCAAAAACATC	Oleastro et al., 2006
Ar (R1-jhp0650/HP0711)	ATTCCAGCGCCTAATGGAC	Oleastro et al., 2006
Bf (F1-jhp0869/HP0935)	AAGAGGATTGCGTGGTGGAGTTG	Oleastro et al., 2006
Br (R1-jhp0871/HP0936)	GGGTTGCCTTTGGGCTTGGA	Oleastro et al., 2006
**qRT-PCR PRIMERS**		
G27_16S RT-F	ATGGATGCTAGTTGTTGGAGGGCT	Gilbreath et al., 2012
G27_16S RT-R	TTAAACCACATGCTCCACCGCTTG	Gilbreath et al., 2012
G27homB FWD Set 4	CCCGGAGAGGCATTTGATAG	This study
G27homB REV Set 4	ACACCCTTTGCGTGTTAGT	This study
**EMSA PRIMERS**		
rpoB EMSA-F	CCAAAGAGGGTAAAGAGAGCG	Carpenter et al., 2009
rpoB EMSA-R	CCTCTCCATCGCTTCTCTAAC	Carpenter et al., 2009
hp0166EMSA_5′	AAAACGATCAAAGGAATTGT	This study
hp0166EMSA_3′	CAATTAACTCCTTCAATGAT	This study
EMSA_G27F	CGCTAAGTAAAGCGCTTTTTAG	This study
GFPfuse_AR	CGCGCTCTAGAGGTTATCTCTTTTTAGTTTATAGTG	This study

A *Regions of overlap sequence for SOE PCR are underlined*.

B *Restriction sites are italicized; lower case nucleotides are nonspecific nucleotides added to enhance restriction digest*.

### Construction of G27 Δ*homB*

All isogenic mutant constructs and strains were created using the *H. pylori* G27 parental strain (Baltrus et al., 2009). The *homB* gene in locus A (HPG27_667) of G27 was replaced with *kan-sacB*. The *kan-sacB* cassette was amplified with primers K4A and PFA3 from pDSM3 (Copass et al., 1997); primer sequences used in this study can be found in Table 2. The *kan-sacB* cassette was then fused to the up-stream region of G27 Locus A (amplified with LocA_UpF/LocA_UpR) and the downstream region of G27 Locus A (amplified with LocA_DnF/LocA_DnR) by SOE PCR (Table 2). The full construct was then amplified with FideliTaq PCR Master Mix (USB) and cloned into pGEM T-easy. Transformants were detected by blue-white screening and confirmed by PCR and Kan^R^. One colony was archived as DSM1530. Wild-type G27 (DSM1) was transformed with pDSM1530, and transformants were selected on HBA Kan^25^, confirmed by PCR, and one colony was archived as DSM1531. To create a clean deletion for use as the background for multiple knockout mutations, pDSM1530 was digested with XbaI and XhoI to remove *kan-sacB* but leave the Locus A flanking sequence. The up and downstream regions of *hom* locus A were then ligated together to create an empty *hom* locus A. The ligation product was transformed into chemically competent Top10 *E. coli*, and transformants were selected on LB+Amp (100 μg/mL). PCR and sequencing were used to confirm the empty locus construct, and one colony was archived as DSM 1670. DSM1531 was transformed with pDSM1670 to create a markerless *hom* deletion in the G27 strain background. Transformants were selected for Suc^R^ and then screened for Kan^S^. Suc^R^Kan^S^ colonies were then confirmed by PCR. Presence of an approximately 1 kB band following PCR amplification with LocA_UpF and LocA_DnR showed that *kan-sacB* had been eliminated, and the upstream and downstream regions of locus A were now fused. One *G27*Δ*homB* colony was selected and archived as DSM1442.

### Construction of *arsRS* Δ*homB* double mutant strains

Both Δ*arsS* Δ*homB* and ArsR-D52N Δ*homB* were constructed in the DSM1442 strain background. To construct G27Δ*arsS* Δ*homB*, DSM1442 was transformed with pDSM920, which carries a Δ*arsS*::*kan* cassette. Transformants were selected on HBA+ Kan25. The *arsS* deletion was confirmed by PCR and sequencing with HP0165_del_ver_F and HP0165_del_ver_F. In addition, LocA_UpF and LocA_DnR were used to ensure the Δ*homB* deletion remained. One isolate was archived as DSM1443.

The G27 ArsR-D52N Δ*homB* strain was constructed in a similar manner. First, The ArsR-D52N Kan^R^ SOE cassette from DSM1446 was PCR amplified with Up1F and Dn4R (Table 2). The PCR product was used to transform DSM1442. Colonies were selected on HBA+Kan^25^. The D52N mutation in ArsR was confirmed by PCR amplification of *arsR* followed by ApoI digestion of the amplicon. One colony with the correct digestion pattern was confirmed by sequencing and archived as DSM1543.

### Construction of *homB* over expression strains

Plasmids containing *homB* fused to the *ureA* or *flaA* promoters were generated to assess the effect of increased *homB* expression on biofilm formation. These plasmid were constructed using the pTM117 plasmid described by Carpenter et al. (2007). First, pDSM463 (pTM117+*ureA* promoter) was digested with XbaI and PstI to drop out the 730 bp GFP gene. *homB* was amplified from G27 with G27*homB*_FXbaI and G27*homB*_RPstI (Table 2). The *homB* amplicon was digested with PstI and XbaI and was then ligated with the similarly digested pDSM463. The ligation was transformed into Top10 *E. coli* and transformants were selected on LB+Kan^25^. Kan^R^ colonies were screened by PCR for the presence of *homB*. Upon confirmation, one colony was archived as DSM1532. pDSM1532 (pUreA-*homB*) was used to transform *H. pylori* G27 (DSM1) and transformants were selected on HBA+Kan^25^. *homB* over-expression was confirmed by qRT-PCR (primers listed in Table 2) for several Kan^R^ colonies. Similar levels of *homB* expression were observed among all tested colonies (data not shown) and one strain was archived as DSM1533 (G27 pUreA-*homB)*. To construct the p*flaA-homB* vector, p*flaA* was first amplified with G27pflaA_FKpnI and G27pflaA_RXbaI from the pDSM3 plasmid (Table 2). The amplicon and pDSM1532 were digested with KpnI and XbaI. Digestion of pDSM1532 resulted in the release of the *ureA* promoter, which was then replaced with the similarly digested *flaA* promoter. Ligation products were transformed into Top10 *E. coli* and selected for on LB+Kan^25^. Kan^R^ colonies were screened for the correct promoter by PCR and sequencing. One correct colony was archived as DSM1538. DSM1 was transformed with the p*flaA-homB* vector, pDSM1538, and the resulting transformant was archived as DSM1539 (G27 pFlaA-*homB*). Similarly, pDSM215, pDSM1532, and pDSM1538 were transformed into G27Δ*homB* (DSM1442) and a single transformant for each was archived: DSM1547 (G27Δ*homB* pTM117), DSM1546 (G27Δ *homB* pUreA-*homB*) and DSM1540 (G27Δ *homB* pFlaA-*homB*), respectively. Plasmid and strain descriptions can be found in Table 1.

### *H. pylori* growth conditions for RNA isolation

Briefly, bacterial cultures were patched from freezer stocks onto HBA plates. After 48 h of plate growth, sterile swabs were used to resuspended bacteria in 5 mL of *H. pylori* liquid media. After 24 h, cultures were standardized to an OD_600_ of 0.05 (OD control culture) in fresh liquid media and allowed to grow for a specified length of time depending on the experiment. To analyze growth dependent expression, one 60 mL OD control culture were separated into 2 separate 125 mL flasks, each containing 30 mL, and cells were collected from alternating flasks at each indicated time point. At each time point, 5 mL of bacterial culture was removed, captured on 0.45 μM cellulose nitrate membrane filter (GE Healthcare), and flash frozen in liquid nitrogen. Flash frozen filters were stored at −80°C until RNA preparation.

Shock experiments were carried out as previously described (Merrell et al., 2003; Carpenter et al., 2015). Briefly, OD control cultures were prepared in a similar manner as described above. 10 mL OD control cultures were collected after 20 h of growth, and separated into two equal portions. One portion was immediately collected on a filter and represented the T_0_ pre-shock sample. The second portion was resuspended in 5 mL of media, which represented one of three environmental shocks: low pH (pH 5), excess nickel (10 μM NiSO4; Sigma-Aldrich), or limited iron [200 μM 2-2′-dipyridyl (DPP)]. pH shock and excess nickel were maintained for 90 min, and iron limitation was carried out for 60 min. All cultures were incubated under microaerobic conditions with shaking at 100 rpm as described under the growth condition section. After the respective incubation period, cultures were collected by the filter method, flash frozen and stored at −80°C.

To examine *homB* expression in biofilm and planktonic cells, biofilms were cultured as previously described; the method is detailed below and described in Servetas et al. (Servetas et al., 2016). Samples were collected for RNA isolation after 24, 48, and 72 h of biofilm growth. At each time point the biofilm and planktonic cells were processed separately. Planktonic cells from three technical replicate wells were pooled, harvested by filtration, and stored at −80°C. Following removal of planktonic cells, each well was washed three times with 2 mL of warm liquid media. After washing, 1.5 mLs of TRIzol reagent (Gibco/BRL) was added to the well, and samples were incubated for 20 min at room temperature to lyse the biofilm cells. The TRIzol from three wells was pooled and stored at −80°C. A total of 4–5 biologically independent biofilm/planktonic samples were cultured and analyzed.

### RNA isolation, cDNA synthesis and quantitative real-time PCR

RNA isolation was conducted as previously described (Thompson et al., 2003). RNA integrity was determined by visualization on a 2% agarose gel. cDNA synthesis and Quantitative Reverse-Transcription PCR (qRT-PCR) were conducted as previously described with a few modifications (Gilbreath et al., 2012). Each cDNA synthesis reaction was carried out using the Quantitect reverse transcriptase kit (Qiagen) and 500 ng of RNA. A control reaction, to ensure there was no genomic DNA contamination, was carried out for each sample by excluding the reverse transcriptase enzyme (NoRT). Following cDNA synthesis, qRT-PCR was performed using an intercalating dye, SYBR green, for *homB* expression and the 16S rRNA internal reference gene. qRT-PCR primers can be found in Table 2. The qRT-PCR reaction mixture was comprised of 1x SYBR green RT-PCR master mix, 3 pmol of each forward and reverse primer, and 1 μl of template (cDNA or the NoRT control). Reactions were brought to a final volume of 20 μl with DNase free water (IDT). The following 3-step cycling protocol was used: a 10 min hold at 95°C (initial denature) was followed by 40 cycles of 10 s at 95°C (denature), 10 s at 58°C (annealing), and 10 s at 72°C (extension). SYBR green fluorescence was measured at the end of the extension step. Relative gene expression of each gene compared to the 16S internal control was calculated by the 2^−Δ*CT*^ method; relative fold difference in gene expression was calculated using the 2^−ΔΔ*CT*^ method. Gene expression studies represent a minimum of three biologically independent experiments.

### Purification of ArsR

Recombinant ArsR (rArsR) was purified from DSM1177 (Harvey et al., 2014). ArsR expression from DSM1177 was induced as previously described (Harvey et al., 2014). Briefly, a 20 mL culture of DSM1177 was grown overnight in Luria-Bertani (LB) (Difco) plus Amp^100^ (100 μg/mL) and Kan^20^ (20 μg/mL) broth. This culture was subcultured 1:50 into 500 mL of prewarmed LB containing Amp^100^ and Kan^20^, and grown with shaking (200 rpm) at 37°C until the OD_600_ reached between 0.5 and 0.7. At this point, expression was induced using 1 mM isopropyl-β-D-thiogalactopyranoside (IPTG) and the culture was placed in a 28°C incubator and grown overnight with shaking at 200 rpm. Following overnight culture, the 500 mL culture was pelleted in two 250 mL volumes. rArsR purification was carried out as previously published (Wen et al., 2006) with several modifications. The pellet was resuspended in native binding buffer (50 mM NaH_2_PO_4_, 500 mM NaCl, 10 mM Tris-HCl, pH 8.0) and stored at −80°C until the purification process. Pellets were thawed on ice, treated with protease inhibitor tablets (IDT) and DNase I (Sigma), and then lysed by French press. Lysate were spun twice to remove cellular debris before loading to a Ni-NTA column (Qiagen) for purification by gravity flow. Approximately 1 mL of Ni-NTA (0.5 mL packed column volume) was used to prepare a column according to the manufacturer directions. The lysates were added to the column and then the flow through was re-loaded to the packed bed volume. The column was then washed sequentially with native binding buffer containing increasing concentrations of imidazole: 40 mL 10 mM imidazole, 40 mL of 20 mM imidazole, 10 mL 30 mM and 10 mL 40 mM imidazole. rArsR was then eluted using 4 mLs of 50 mM imidazole followed by 4 mL 100 mM imidazole. Fractions from wash and elution steps were checked on SDS-Page gel by total protein staining and Western blot analysis to assess purity. The 50 mM fraction was pooled, concentrated and buffer exchanged into native binding buffer using Amicon 10 kD MWCO filtration devices and then quantified. The final concentration of rArsR was calculated to be 327.3 μg/mL.

### ArsR electromobility shift assay (EMSA)

The promoter regions for *H. pylori* G27 *homB, arsR* (positive control), and *rpoB* (negative control) were amplified using the primers indicated in Table 2. Promoter region amplicons were end labeled with [^32^P] ATP (Perkin Elmer) as previously described (Gancz et al., 2006). Briefly, 150 ng of each amplicon was combined with 5 μl of [^32^P] ATP and T4 polynucleotide kinase (NEB). To remove unincorporated nucleotides, reactions were cleaned with the MinElute Reaction Clean-up Kit (Qiagen) and eluted with 30 μL EB. 60 μL of EMSA binding buffer (25 mM NaPO4 (pH 7), 150 mM NaCl, 0.1 mM MgSO4, 1 mM DTT, 0.6 ug/mL BSA, 200 ng/μl salmon sperm, 4% glycerol) was then added to the labeled templates.

Prior to each EMSA trial, ArsR was phosphorylated *in vitro* using a lithium potassium acetyl phosphate high energy phosphate donor (Sigma-Aldrich) (Carpenter et al., 2015; Kinoshita-Kikuta et al., 2015). rArsR was diluted 1:2 in phosphorylation buffer (0.3 M Tris-HCl, 50 mM KCl, 10 mM MgCl2, 1 mM DTT, and 40 mM acetyl phosphate). The phosphorylation was carried out at 25°C for 60 min. Immediately following phosphorylation, ArsR~P was serially diluted in EMSA binding buffer and combined with the appropriate EMSA template. For each template, in addition to a no protein control reaction, a cold competition reaction in which 2x unlabeled promoter template was added in addition to labeled template was conducted. All reactions were allowed to incubate for 30 min at room temp. Following incubation, templates were loaded onto 6% DNA retardation gels (Invitrogen) and run for 1 h at 100 V in 0.5X TBE. Gels were then exposed to phosphor screens overnight and scanned on a Storm 860 Scanner (GE Healthcare), followed by analysis using ImageJ64 (NIH).

### Biofilm and SEM analyses

Biofilm assays were conducted as previously described (Servetas et al., 2016). Briefly, biofilms were grown in a 24-well tissue culture treated plate. Cultures were added to each well to an OD_600_ of 0.1 in 1 mL of HBA liquid media. Biofilms were allowed to develop for 24, 48, and 72 h. At each time point, planktonic cells were aspirated, wells were washed twice with PBS, biofilms were fixed with methanol, and then stained with crystal violet. Crystal violet was rinsed away 3x with distilled water and then solubilized to assess relative quantity of biofilm. Solubilized crystal violet was read at OD_590_. Data shown represent a minimum of 3 biological replicates. Scanning electron microscopy (SEM) assays were performed as previously described (Servetas et al., 2016). Fresh liquid media in 12 or 24 well plates was inoculated as a 1:10 dilution with cells from overnight cultures (0.3–0.7 OD_600_). Cell-cultured treated cover-slips were placed at the bottom of each well to capture biofilm formation. Samples were treated and visualized by SEM as previously described (Gaddy et al., 2009). SEM images are representative of three biological replicates.

### Statistical analysis

*homB* expression over time was analyzed by two-way ANOVA with Tukey's correction. The comparison of *homB* expression between biofilm and planktonic cells was conducted using a paired analysis two-way ANOVA with Sidak's correction for multiple comparisons. The rate of biofilm formation, which is defined as the first time point where the crystal violet quantification was significantly greater than the blank, was determined by a two-way ANOVA with Dunnett's correction. Differences in amount of biofilm formation between strains at each time point were analyzed using a one-way ANOVA with Tukey's correction for multiple comparisons. All analyses were conducted with GraphPad Prism 6.

## Results

### The *homB* promoter region is highly conserved regardless of locus

HomB can be found at two different chromosomal loci (Oleastro et al., 2009a; Servetas et al., 2018). To assess *homB* promoter variability based on the chromosomal locus, we first used BLAST to determine the overall conservation between the *homB* promoter regions available in Genbank. We found that the promoter regions at both loci were highly conserved. For instance, the BLAST analysis using the entire 228 bp intergenic region upstream of *homB* (locus A) from G27 returned matches with complete coverage that ranged from 100 to 91% identify. When this comparison was extended to include strains that carried a *homB* gene at locus B, approximately 85% coverage with identity scores ranging from 99 to 88% were seen. In some rare cases, even when the locus was empty, parts of the *homB* promoter region could still be found. In at least one strain (sequence ID: CP006610.2) although locus B was empty, there was a 136 bp fragment present that showed 91.9% identity to the *homB* promoter region. Overall, at least 178 bps appeared to be well conserved between the promoter of *homB* at locus A and locus B. To better visualize this conservation, the sequences from 30 *homB* promoter regions (approximately 180 bp upstream from the *homB* translational start site) were aligned. The pairwise alignment found a range of 80–100% identity between individual promoters (Supplementary Figure 1, Figure 1). Visualization of the conservation as a weblogo revealed that in addition to regions such as the −10, which is 100% conserved among the 30 loci, there were several other highly conserved regions (Figure 1). This high degree of conservation in the intergenic promoter region regardless of chromosomal locus could suggest that this area is conserved to maintain binding sequences for regulatory proteins.

### *homB* expression is modulated by growth phase and environmental cues

**Figure 2:**
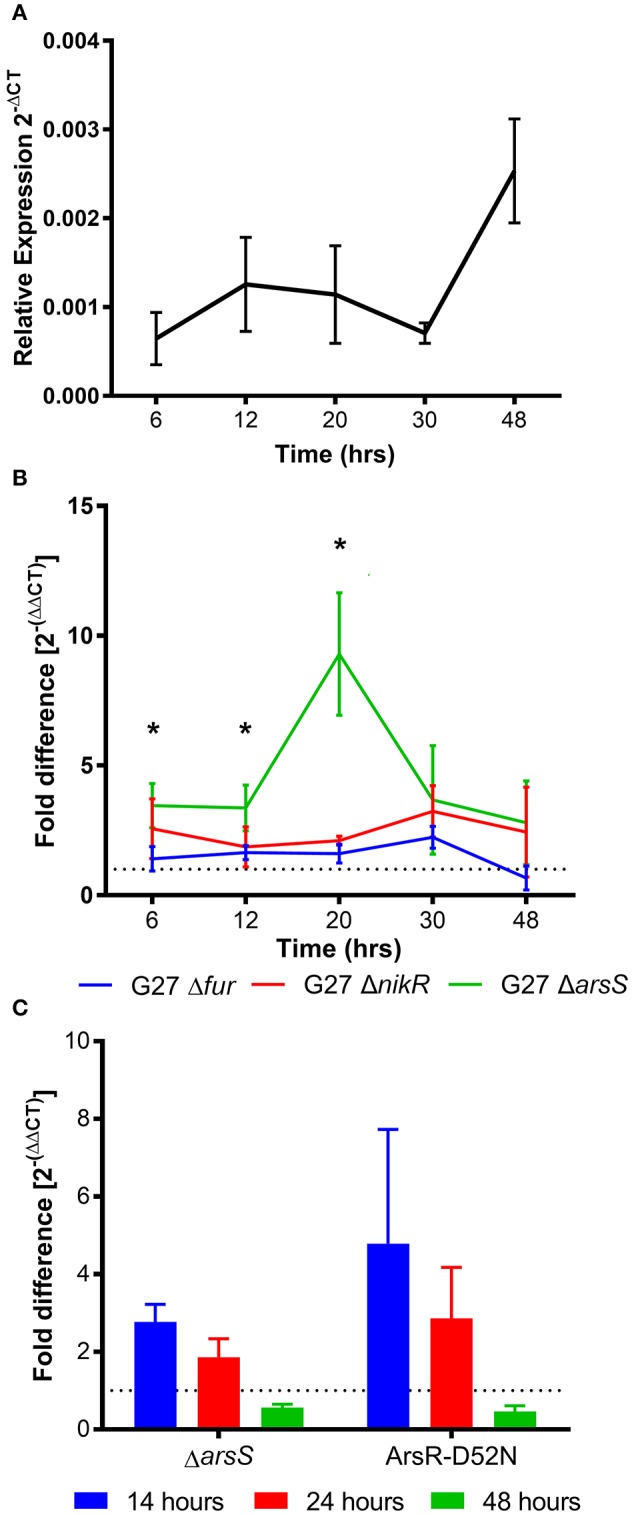
*homB* temporal expression in wild-type and regulator-deficient mutant strains. **(A)** *homB* expression profile in wild-type *Helicobacter pylori* strain, G27. The relative expression of *homB* as compared to the 16S gene was calculated for each time point shown on the x-axis. The solid line is plotted at the mean of 3 biologically independent experiments; errors bars represent the standard error of the mean. **(B)** *homB* expression was monitored over time in strains lacking Fur, NikR, and ArsR. The fold difference between wild-type and each regulatory knockout is plotted over time. The solid lines are plotted at the mean of three biological replicates and errors bars show the standard error of the mean. A dotted line is plotted at 1 to indicate no fold difference in *homB* expression compared to wild-type. An asterisk indicates a significant difference in *homB* expression between wild-type and the indicated regulatory mutant strain at that given time point: ^*^p-value < 0.05. A significant difference in *homB* expression was observed in the Δ*arsS* strain at T6 (*p* = 0.036), T12 (*p* = 0.0477), and T20 (*p* = 0.0182). A significant difference in *homB* expression was observed at T20 in the Δ*nikR* strain (*p* = 0.0114). **(C)** *homB* expression in Δ*arsS* and Ars-D52N mutant strains was assessed after 14, 24, and 48 h. Fold difference at each time point was calculated between the mutants and the wild-type. A dotted line is plotted at 1 to indicate no fold difference. Each bar represents the mean of three biologically independent experiments and errors bars show the standard error of the mean.

There is currently little data regarding the expression of *homB*; however, immunologic data suggests that *homB* is expressed during infection (Oleastro et al., 2008). To explore *homB* expression, qRT-PCR was used to quantify *homB* transcription levels over a standard 48 h *in vitro* growth curve of *H. pylori* G27 (Figure 2A). Relatively low and consistent levels of expression over the first 30 h of growth were observed, followed by an approximately 3-fold increase in expression at 48 h. Although the observed increase at 48 h was not statistically significant, these data suggest that *homB* expression is increased in stationary phase (Figure 2A). Next, we sought to determine what regulatory factor(s) were responsible for modulation of *homB* expression. To this end, *homB* expression was assessed in the absence of each of the following well characterized transcriptional regulators: Fur, NikR, and ArsS (Figure 2B). Fur and NikR both serve as metallo-regulators that respond to changes in iron and nickel, respectively; ArsS, is the sensor component of a two component system, ArsRS, which functions in response to changes in pH. While the pattern of *homB* expression observed in the Δ*fur* background was similar to the wild-type strain (Figure 2B), *homB* expression was increased in both the Δ*nikR* and Δ*arsS* strains throughout the growth curve. Specifically, in the Δ*nikR* strain background, an average of a two-fold increase in *homB* expression was observed at all time-points as compared to wild-type. Furthermore, in the Δ*arsS* strain, transcript levels of *homB* were elevated between four- and ten-fold in comparison to wild-type over the course of the experiment. Furthermore, where wild-type *homB* expression peaked at 48 h, deletion of *arsS* resulted in a shift in maximal *homB* expression from the stationary phase to mid-log phase (Figure 2B). In the Δ*arsS* strain, a significant increase in expression was observed at 20 h compared to 6 h (*p* = 0.0086), 12 h (*p* = 0.0075), 30 h (*p* = 0.0295), and 48 h (*p* = 0.003) (Figure 2B). Together, these data suggest that NikR and the ArsRS two-component system play roles in regulation of *homB* expression.

**Figure 3:**
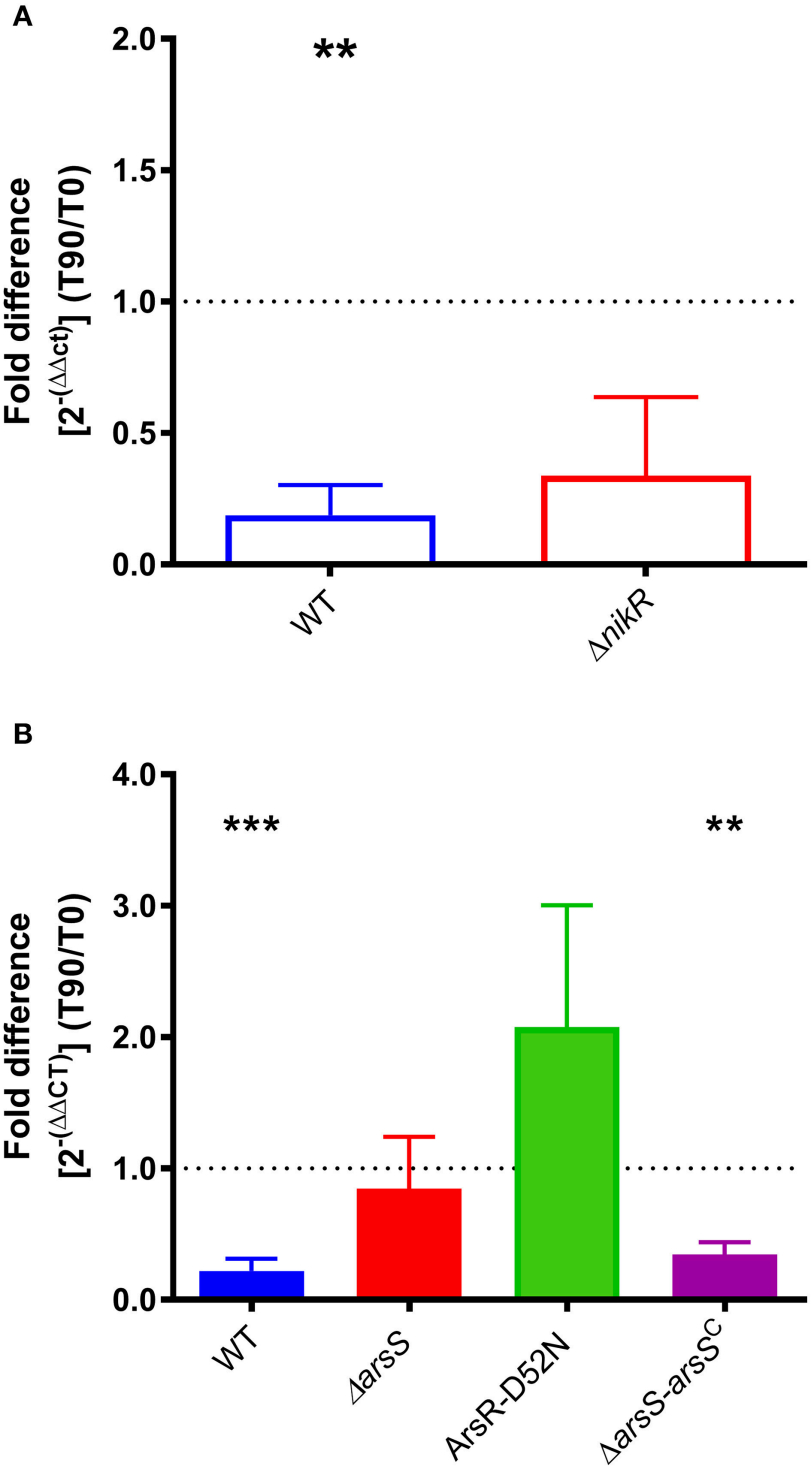
*homB* expression in response to environmental stimuli. **(A)** The expression of *homB* was evaluated in wild-type and Δ*nikR* following a 90 min exposure to 10 μM Ni^2+^. The fold difference between pre-shock (T0) and post-shock (T90) was calculated, and the mean fold difference of three biological replicates was plotted. The error bars show the standard error of the mean. A dotted line is plotted at 1 to indicate no fold difference in expression between pre-shock and post-shock expression. A significant decrease in expression was observed in the wild-type strain following exposure to 10 μM Ni^2+^ (*p* = 0.0047). **(B)** The expression of *homB* was evaluated in response to a 90 min shock at pH 5 in wild-type, Δ*arsS*, ArsR-D52N, and Δ*arsR-arsS*^*C*^. The fold difference between pre-shock (T0) and post-shock (T90) expression was calculated and plotted. A dotted line is plotted at 1 to indicate no fold difference *homB* expression following pH shock. Each bar represents the mean of a minimum of three biological replicates and error bars display the standard error of the mean. A significant decrease in *homB* expression was observed in wild-type (*p* = 0.0004) and Δ*arsR-arsS*^*C*^ (*p* = 0.0021). Asterisks indicate significant changes in *homB* expression (^**^p < 0.01, ^***^p < 0.001).

Given the increase in *homB* expression observed in the Δ*nikR* and Δ*arsS* strain backgrounds, we next asked how *homB* expression changed in response to the environmental cues that are sensed by these regulators, nickel and pH, respectively. In wild-type *H. pylori*, a significant, approximately five-fold decrease in *homB* expression following a 90 min shock with 10 μM Ni^2+^ was observed (Figure 3A). Unexpectedly, in the Δ*nikR* strain background a similar decrease occurred. If NikR were responsible for the decrease in *homB* expression, one would expect *homB* expression in the Δ*nikR* strain to remain unaffected by the presence of nickel; however, no difference in *homB* expression between wild-type and the Δ*nikR* strain was observed (*p* = 0.3081) suggesting *homB* is still regulated even in the absence of NikR. Together, these data suggest that the decrease in *homB* expression in response to excess nickel is NikR-independent. In regards to pH, when wild-type *H. pylori* was subjected to a pH of 5 for 90 min, a 10-fold decrease in *homB* expression as compared to pH 7 was observed (Figure 3B). Conversely, when the Δ*arsS* strain was similarly exposed to pH 5, little to no decrease in *homB* expression was seen. This finding suggests that *homB* repression at low pH is ArsS-dependent (Figure 3B).

As mentioned, ArsS is the sensor kinase portion of the two-component system, ArsRS. In response to decreasing pH, ArsS phosphorylates ArsR and ArsR~P can then regulate gene expression. Ideally, this regulatory cascade would be assessed by knocking out *arsR*; however, this gene is essential and deletion of *arsS* is often used as a proxy. Another means of assessing this regulatory pathway is via the use of a non-phosphorylatable ArsR-D52N mutant strain; this strain is still viable but ArsR is unable to be phosphorylated in response to low pH. Given the dramatic effect that loss of ArsS had on *homB* expression, we next tested the same conditions using an ArsR-D52N mutant strain. In agreement with the increase in *homB* expression seen at early time points in the Δ*arsS* strain background, an increase in *homB* expression in the ArsR-D52N mutant strain was also detected during log phase (Figure 2C). Additionally, when ArsR-D52N was exposed to pH 5 shock, there was no decrease in *homB* expression (Figure 3B). These data further support the hypothesis that *homB* expression is repressed by ArsRS in response to low pH. Also of note, when *arsS* was expressed *in trans* (complemented) in the Δ*arsS* strain background (Δ*arsS-arsS*^*c*^), decreased *homB* expression in response to low pH was restored.

**Figure 4:**
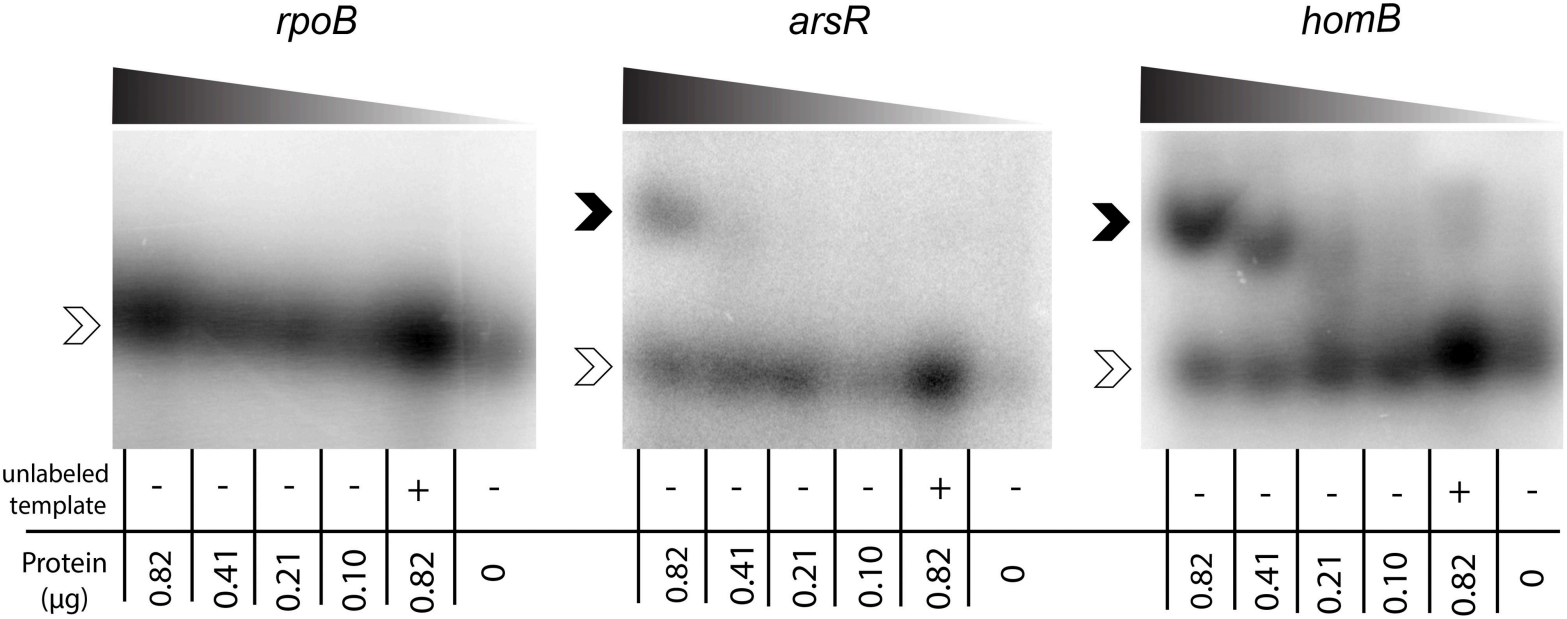
ArsR EMSA. rArsR was purified and phosphorylated *in vitro*. Binding to the *rpoB* promoter (negative control), *arsR* promoter (positive control), and *homB* promoter were assessed. Protein was loaded in decreasing concentrations from left to right across the gel (0.82–0.10 μg) and is represented by the shaded wedge above each gel. A competition reaction with 2x unlabeled template and 0.82 μg protein was run in the lane second from the right of each gel and a no protein control was run in the right most lane. Open arrowheads indicate unbound DNA template, and filled arrowheads indicate DNA template bound to rArsR~P.

To determine if ArsR was directly regulating *homB* expression via interactions with the gene's promoter, ArsR-promoter interactions were assessed by EMSA. Given that ArsR is auto-regulatory, we used the *arsR* promoter region as a positive control (Dietz et al., 2002). In addition, the promoter of *rpoB* was used as a negative control to show absence of non-specific binding. As shown in Figure 4, a clear shift in the banding pattern was observed for both the *arsR* and *homB* promoter regions (Figure 4). In comparison, no shift was observed for *rpoB* (Figure 4). Of note, a shift in the *homB* promoter was observed even at low concentrations of rArsR~P, whereas the interaction with the *arsR* promoter region was only evident at higher concentrations. Finally, interaction with the *arsR* and *homB* promoter regions was specific as the addition of excess unlabeled promoter fragment was able to shift the band migration to the same area as the no protein control.

### *homB* contributes to hyper biofilm formation in ArsRS mutant strains

**Figure 5:**
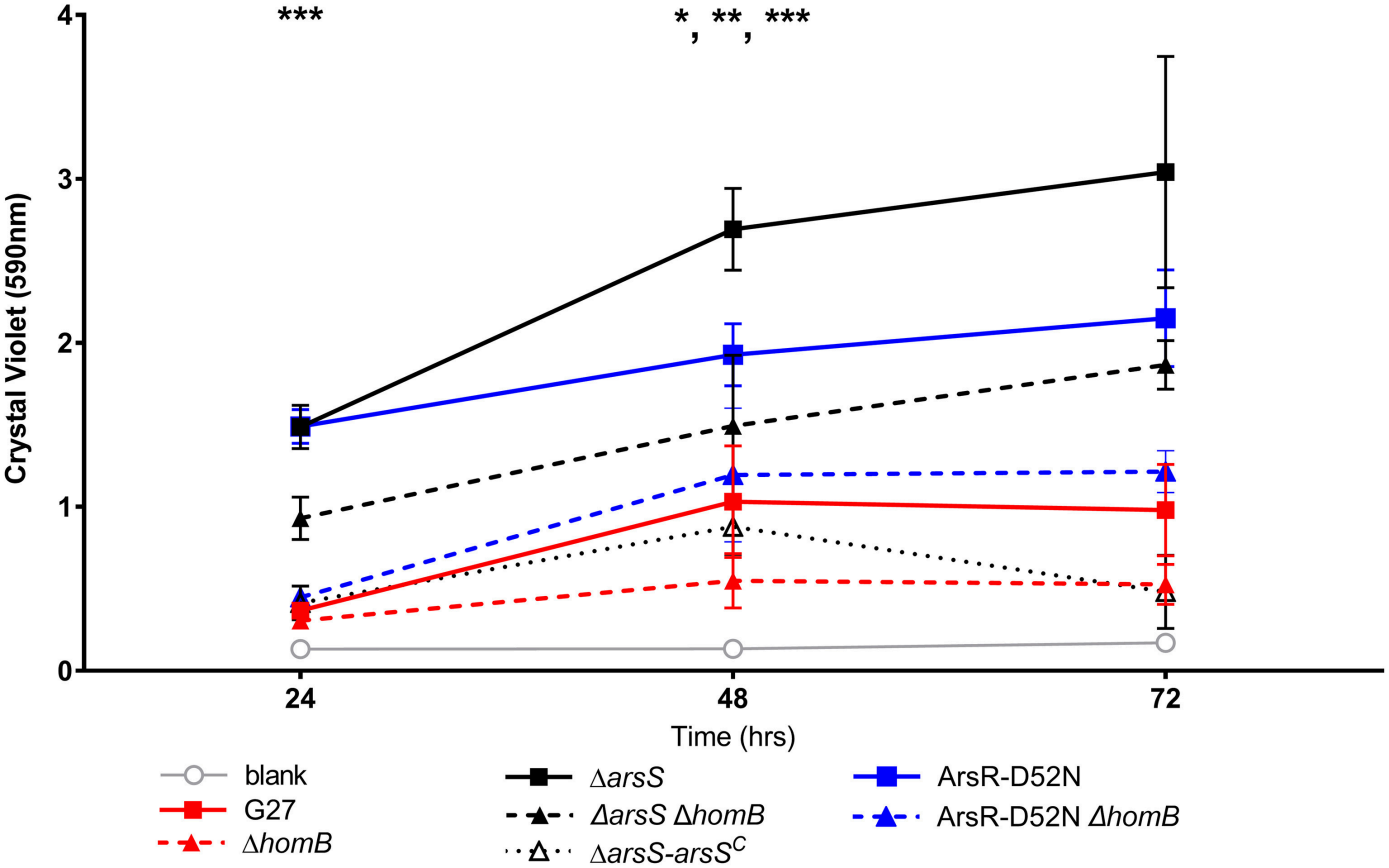
Crystal violet biofilm quantification. Solubilized crystal violet stain was read at an OD590 and plotted over time. The mean absorbance for a minimum of three biologically independent replicates is indicated by each line. For all data sets, error bars show the standard error of the mean. An asterisk indicates significant biofilm formation compared to the blank at a given timepoint (^*^p < 0.05, ^**^p < 0.01, ^***^p < 0.001). By 24 h, significant biofilm formation was observed for Δ*arsS* vs. blank (*p* = 0.0005) and ArsR-D52N vs. blank (*p* = 0.0005); significant biofilm formation was observed by 48 h for wild-type vs. blank (*p* = 0.0350), Δ*arsS*Δ*homB* vs. blank (*p* = 0.0005), and ArsR-D52NΔ*homB* vs. blank (*p* = 0.0088).

We recently showed that strains with a mutation in either portion of the ArsRS two-component system (Δ*arsS* or ArsR-D52N) form significantly more biofilm and do so at an enhanced rate as compared to wild-type *H. pylori* (Servetas et al., 2016). Given that *homB* was highly over expressed in both the Δ*arsS* and ArsR-D52N strains (Figure 2C), we reasoned that increased expression of this OMP could contribute to the previously observed biofilm phenotype. Visual analysis of these samples revealed dramatic strain dependent differences in crystal violet staining on the bottom of the wells (Supplementary Figure 2). Furthermore, crystal violet assay quantification of biofilm formation, showed similar results as in our previous studies (Servetas et al., 2016); significant biofilm formation occurred earlier in the Δ*arsS* and ArsR-D52N strains (24 h) as compared to wild-type (48 h) (Figure 5). Deletion of *homB* from the Δ*arsS* or ArsR-D52N strains negated the early biofilm phenotype; significant biofilm formation was not seen until 48 h in the double mutant strains (Figure 5). In addition, by 48 and 72 h, Δ*arsS* and ArsR-D52N formed significantly more biofilm than wild-type, respectively, whereas Δ*arsS* Δ*homB* and ArsR-D52NΔ*homB* did not (Figure 5, Table 3). Overall there was less biofilm formation in all of the Δ*homB* strains as compared to their cognate *homB* positive strains; however, these differences were not always statistically significant (Figure 5, Table 3).

**Table 3:** Statistical analysis of biofilm formation between strains carrying ArsRS mutations.

**Comparison**	***P*-value^A^**	**Summary**
**24 HOURS**		
G27 vs. G27Δ*homB*	>0.9999	ns
G27 vs. G27Δ*arsS*	0.0604	ns
G27 vs. G27ArsR-D52N	0.059	ns
G27 vs. G27Δ*arsS*Δ*homB*	0.7348	ns
G27 vs. G27Δ*homB* ArsR-D52N	>0.9999	ns
G27Δars*S* vs. G27Δ*arsS*Δ*hom*B	0.7416	ns
G27ArsR-D52N vs. G27Δ*homB* ArsR-D52N	0.0973	ns
G27Δ*homB* vs. G27Δ*ars*SΔ*homB*	0.6305	ns
G27Δ*homB* vs. G27Δ*homB* ArsR-D52N	0.9997	ns
**48 HOURS**		
G27 vs. G27Δ*homB*	0.8478	ns
G27 vs. G27Δ*arsS*	0.001	^**^
G27 vs. G27ArsR-D52N	0.2185	ns
G27 vs. G27Δ*arsS*Δ*homB*	0.873	ns
G27 vs. G27Δ*homB* ArsR-D52N	0.9994	ns
G27Δ*arsS* vs. G27Δ*arsS*Δ*homB*	0.0355	^*^
G27ArsR-D52N vs. G27Δ*homB* ArsR-D52N	0.4449	ns
G27Δ*homB* vs. G27Δ*arsS*Δ*homB*	0.1702	ns
G27Δ*homB* vs. G27Δ*homB* ArsR-D52N	0.5948	ns
**72 HOURS**		
G27 vs. G27Δ*homB*	0.8825	ns
G27 vs. G27Δ*arsS*	< 0.0001	^****^
G27 vs. G27ArsR-D52N	0.0434	^*^
G27 vs. G27Δ*arsS*Δ*homB*	0.2289	ns
G27 vs. G27Δ*homB* ArsR-D52N	0.9953	ns
G27ΔarsS vs. G27Δ*arsS*Δ*homB*	0.0419	^*^
G27ArsR-D52N vs. G27Δ*homB* ArsR-D52N	0.1782	ns
G27Δ*homB* vs. G27Δ*arsS*Δ*homB*	0.0132	^*^
G27Δ*homB* vs. G27Δ*homB* ArsR-D52N	0.5214	ns

A ns: p > 0.05;

* p ≤ 0.05;

** p ≤ 0.01;

**** *p ≤ 0.0001*.

### *homB* expression is increased during early biofilm formation and over expression enhances biofilm formation

**Figure 6:**
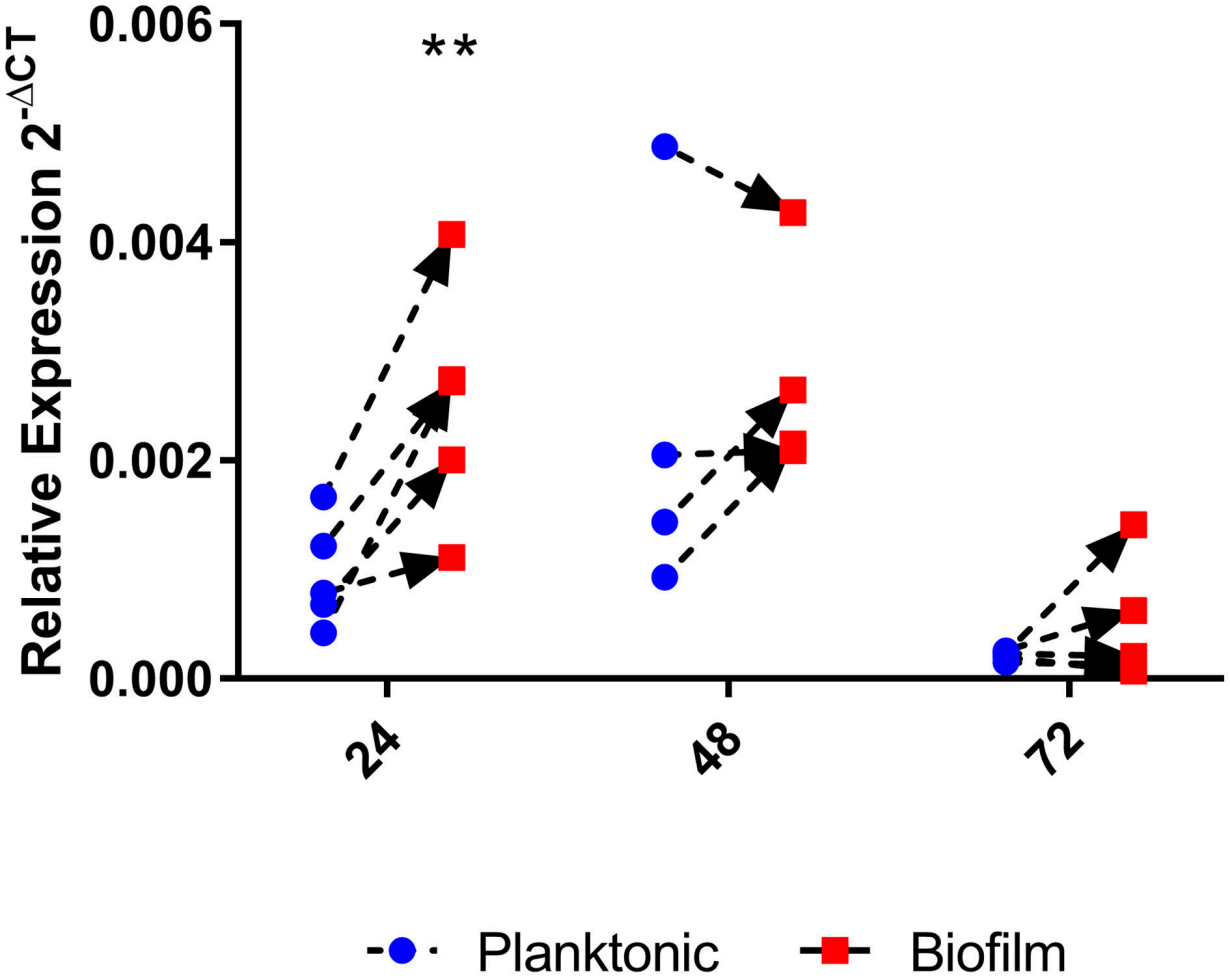
*homB* expression in biofilm cells compared to planktonic cells. The expression of *homB* was evaluated in biofilm cells as compared to planktonically growing cells from the same culture. The relative expression of *homB* as compared to the 16S gene was calculated for each time point and is shown on the x-axis. A minimum of 4 biological replicates were conducted for each time point; dotted lines with arrows connect paired samples. A significant difference in *homB* expression between biofilm and planktonic derived cells was observed at 24 h (*p* = 0.0023) as indicated by the ^**^.

Given the above results that indicated that *homB* contributes to hyper-biofilm formation, we next asked if *homB* expression was altered in wild-type biofilm cells as compared to planktonic cells. To assess this, we compared *homB* expression in biofilm associated bacteria to the planktonic bacteria from the same culture. At 24 h, we observed a significant increase in *homB* expression in cells within the biofilm as compared to the planktonic cells (Figure 6); by 48 h, this difference was no longer observed. These findings may suggest that increased *homB* expression helps to establish early biofilm formation; cells expressing higher levels of *homB* may adhere and initiate the biofilm process. Of note, significant biofilm formation is not observed with wild-type cells until; missing space between cell and until 48 h, which is when the disparity in *homB* expression between biofilm and planktonic cells is no longer observed (Figures 5, 6).

**Figure 7:**
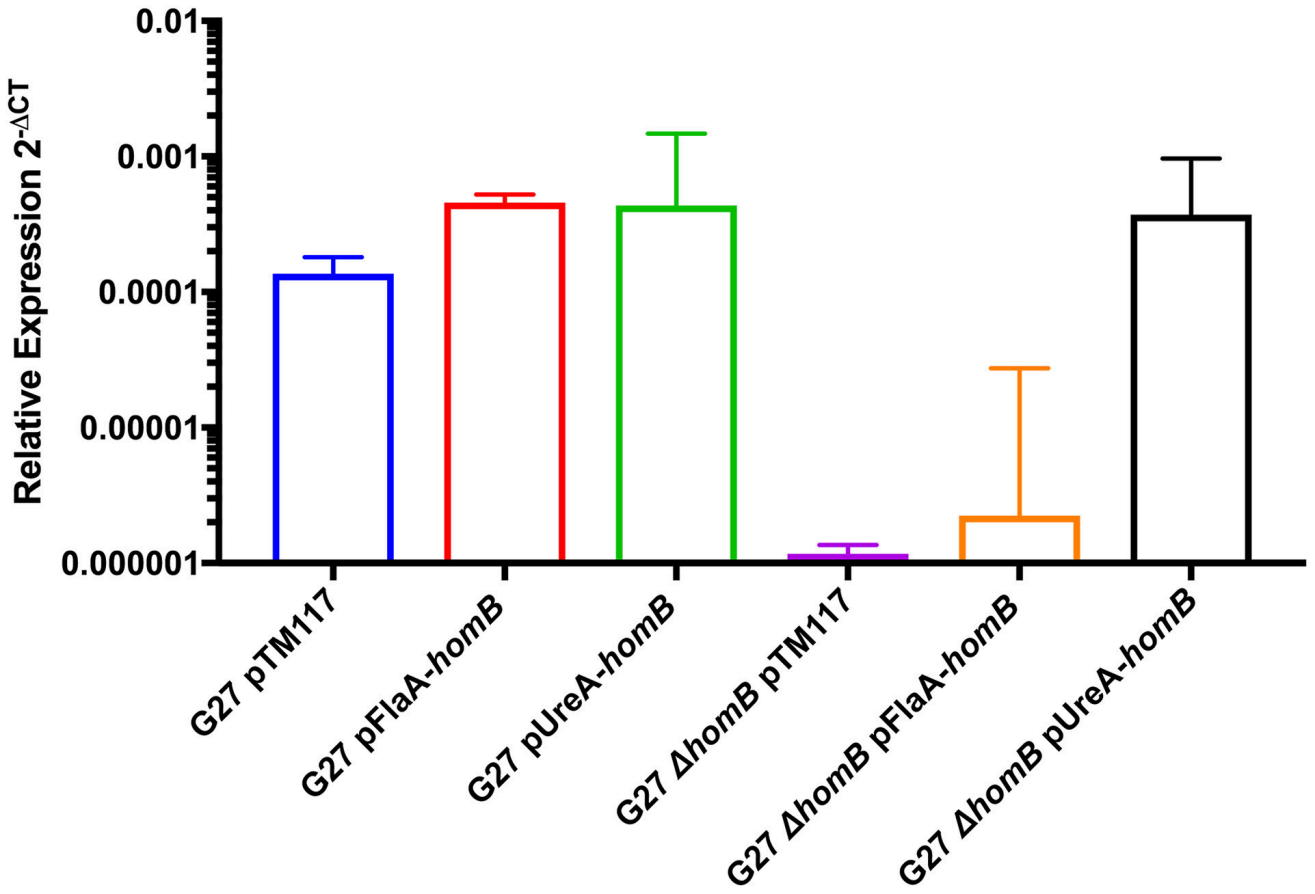
Relative *homB* expression driven by the *flaA* and *ureA* promoters. *homB* expression was assessed after 20 h of growth by qRT-PCR for the strains indicated above. The relative expression of *homB* as compared to the 16S gene was calculated for each time point shown on the x-axis. Each bar displays the mean of 3 biologically independent experiments; errors bars represent the standard error of the mean. Compared to G27Δ*homB* pTM117, a significant increase in *homB* expression was observed in all strains except for G27Δ*homB* pFlaA-*homB*.

Thus far, we have shown that *homB* is regulated by the ArsRS two component system and that loss of ArsRS regulation leads to an increase in *homB* expression and a higher level of *homB* expression at earlier time points (Figure 2). Furthermore deletion of *homB* reversed the hyper-biofilm phenotype in the ArsR-D52N and Δ*arsS* backgrounds. Given these observations, we next asked if increased *homB* expression in either a wild-type or Δ*homB* strain was sufficient to induce the enhanced biofilm formation that was observed in the ArsR-D52N and Δ*arsS* mutant strains. To accomplish this goal, constructs where *homB* expression was driven by either the *flaA* or *ureA* promoters were generated and moved into G27 and G27 Δ*homB*. As expected, both the *flaA* and *ureA* promoter fusions resulted in increased *homB* expression as compared to strains carrying the vector control (Figure 7). Of note, despite the fact that the *ureA* promoter is known to be highly active in *H. pylori* (Bauerfeind et al., 1997), both promoters resulted in comparable levels of *homB* expression in the WT background. In contrast, higher levels of *homB* expression were observed in G27 Δ*homB* pUreA-*homB* compared to G27 Δ*homB* pFlaA-*homB* (Figure 7). Furthermore, despite the lack of a native copy of *homB*, the levels of *homB* expression in the G27 Δ*homB* pUreA-*homB* were similar to the levels of *homB* expression seen in G27 pUreA-*homB*. These data suggest that there is a maximum level of *homB* transcription that can be tolerated in *H. pylori*.

**Figure 8:**
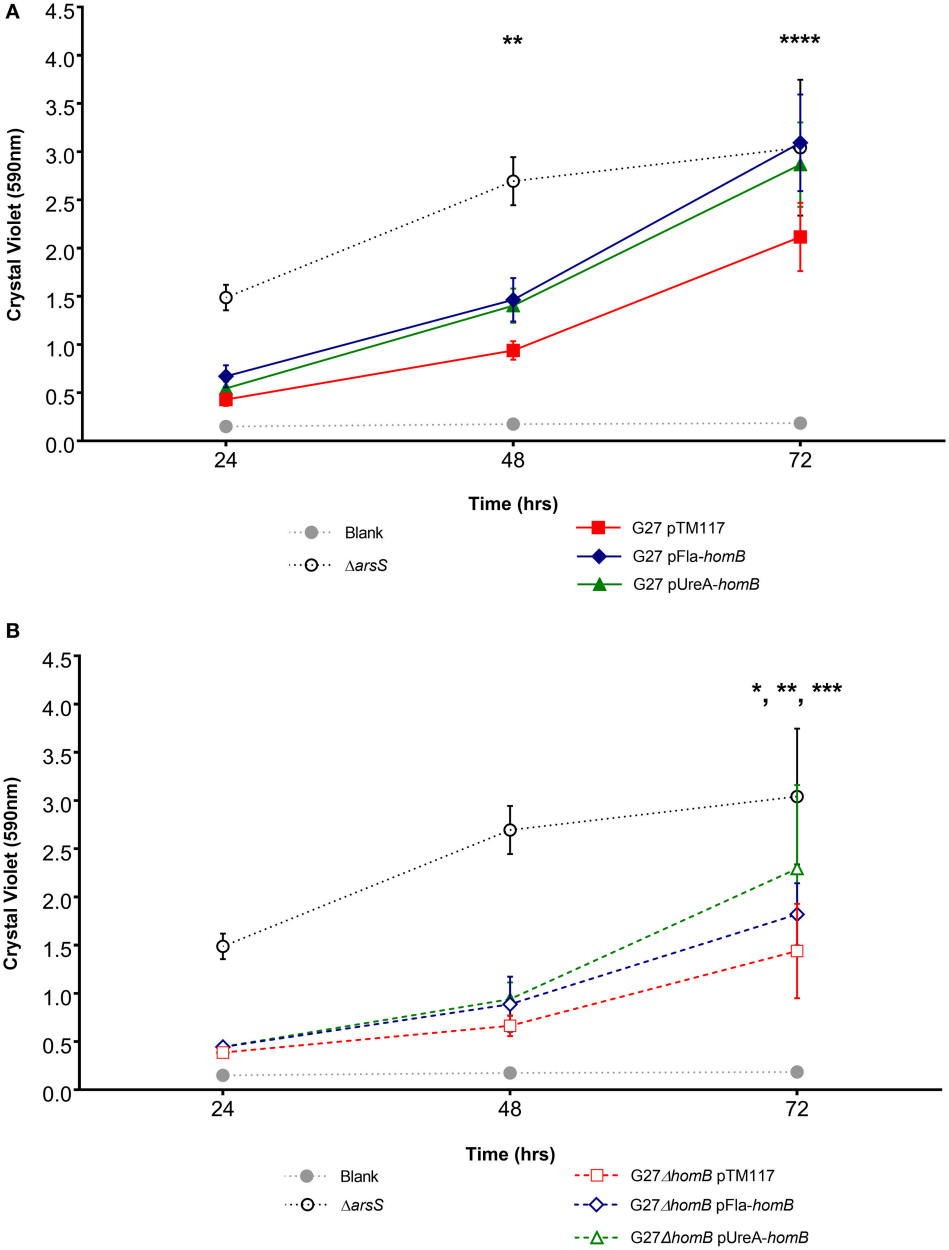
Biofilm formation in p*homB* strains. Biofilm formation was assessed using crystal violet staining for **(A)**. wild-type and **(B)** Δ*homB* strains carrying either the empty vector (pTM117), pUreA-*homB*, or pFlaA-*homB*. Biofilm formation in the Δ*arsS* strain is shown for comparison by the dotted black line. An asterisk indicates significant biofilm formation compared to the blank at a given timepoint (^*^p < 0.05, ^**^p < 0.01, ^***^p < 0.001, ^****^p ≤ 0.0001). Significant biofilm formation was observed at 48 h compared to the blank for G27 pUreA-*homB* (*p* = 0.004) and G27 pFlaA-*homB* (*p* = 0.0068). Significant biofilm formation was observed at 72 h compared to the blank for G27 pTM117 (*p* = 0.0001), G27 Δ*homB* pTM117 (*p* = 0.0241), G27 Δ*homB* pUreA-*homB* (*p* = 0.0002), and G27 Δ*homB* pFlaA-*homB* (*p* = 0.0031).

Overall, we were able to obtain strains in which *homB* was expressed between 4-fold to 10-fold higher than the wild-type strain. We first assessed the biofilm formation of these strains in the crystal violet assay and found that the over-expression of *homB* was not sufficient to completely recapitulate the dramatic enhancement of biofilm formation that was observed in the ArsR-D52N or Δ*arsS* strains in this assay (Figure 8). There was a trend toward increased biofilm formation by G27 pFlaA-*homB* and G27 pUreA-*homB* at 48 and 72 h and at 72 h there was significantly more biofilm in the G27 pFlaA-*homB* and G27 pUreA-*homB* as compared to the control (Figure 8A, Table 4). Additionally, both G27 pFlaA-*homB* and G27 pUreA-*homB* displayed significant biofilm formation by 48 h, in comparison to the empty vector control which did not display significant biofilm formation until the 72 h time point. Similarly, in the G27 Δ*homB* background, there was a trend toward increased biofilm formation in the pFlaA-*homB* and pUreA-*homB* containing strains (Figure 8B, Table 4). These data suggest that over expression of *homB* may enhance the overall amount of biofilm formation but, this alone was not sufficient to recapitulate the enhanced biofilm formation seen in this assay when the ArsRS system is mutated.

**Table 4:** Statistical analysis of biofilm formation between strains carrying *homB* expression vectors.

**Comparison**	***P*-value^A^**	**Summary**
**24 HOURS**		
G27 pTM117 vs. G27 pFla-*homB*	ns	0.6489
G27 pTM117 vs. G27 pUreA-*homB*	ns	0.9023
G27Δ*homB* pTM117 vs. G27Δ*homB* pFla-*homB*	ns	0.9853
G27Δ*homB* pTM117 vs. G27Δ*homB* pUreA-*homB*	ns	0.9841
**48 HOURS**		
G27 pTM117 vs. G27 pFla-*homB*	ns	0.168
G27 pTM117 vs. G27 pUreA-*homB*	ns	0.2375
G27Δ*homB* pTM117 vs. G27Δ*homB* pFla-*homB*	ns	0.8074
G27Δ*homB* pTM117 vs. G27Δ*homB* pUreA-*homB*	ns	0.7257
**72 HOURS**		
G27 pTM117 vs. G27 pFla-*homB*	^**^	0.0066
G27 pTM117 vs. G27 pUreA-*homB*	^*^	0.0381
G27Δ*homB* pTM117 vs. G27Δ*homB* pFla-*homB*	ns	0.5599
G27Δ*homB* pTM117 vs. G27Δ*homB* pUreA-*homB*	ns	0.0908

A ns: p > 0.05;

* p ≤ 0.05;

** *p ≤ 0.01*.

### Biofilm SEMs reveal dramatic *homB*-dependent differences in biofilm formation

**Figure 9:**
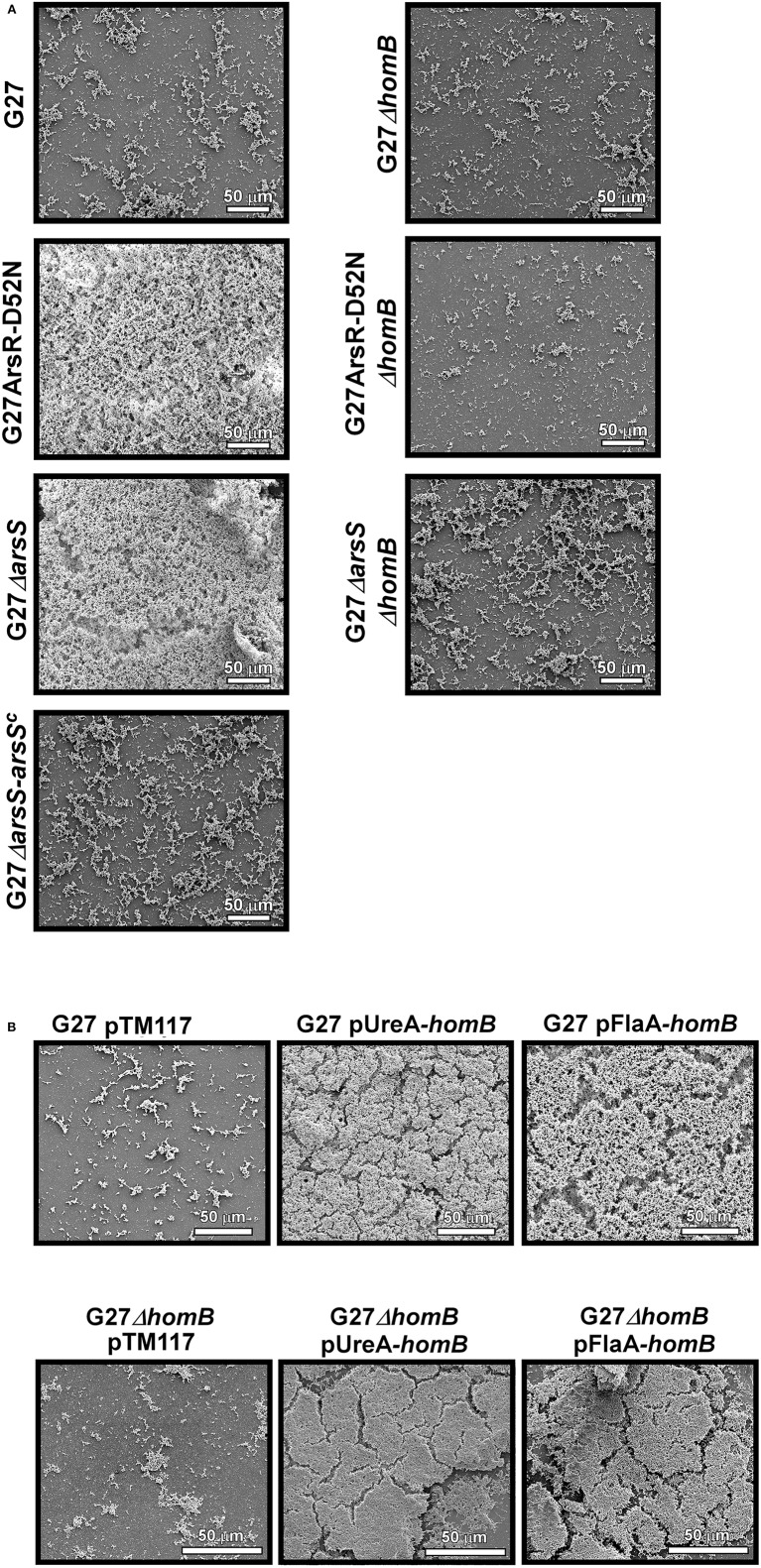
SEM analysis of *H. pylori* biofilm formation. *H. pylori* biofilms were visualized by SEM. **(A)** 1000x SEM images of biofilm formation at 48 h by wild-type G27, Δ*arsS*, and ArsR-D52N as compared to their isogenic Δ*homB* strain derivatives and the *arsS* complemented strain. **(B)** 1000X images of biofilm formation at 48 h by G27 and G27Δ*homB* strains carrying a control vector (pTM117) or vectors containing *homB* under control of the *ureA* or *flaA* promoters.

Given that crystal violet staining is not the most sensitive measure of biofilm formation, and given that there are sometimes apparent visual differences in biofilm staining (Supplementary Figure 2), we next used SEM imaging as a qualitative and semi-quantitative approach to directly visual biofilm formation of the isogenic mutant strains (Figure 9, Supplementary Figure 2). When wild-type *H. pylori* were viewed at the lower magnification, we observed slight biofilm formation and numerous adherent bacteria (Figure 9A). In comparison, less biofilm and adherent bacteria were observed for the Δ*homB* strain (Figure 9A). As expected, striking biofilm formation was observed for both of the ArsRS mutant strains. Deletion of *homB* in both of the strains drastically reduced biofilm formation (Figure 9A). Furthermore, a decrease in biofilm formation was also observed when *arsS* was supplemented *in trans* (Δ*arsS-arsS*^*C*^) (Figure 9A). SEM analysis also revealed a dramatic difference between the empty vector control strain (G27 pTM117) as compared to the *homB* over expression strains (Figure 9B). All strains containing vectors that over expressed *homB* displayed enhanced biofilm formation; these levels were similar to what was seen in the ArsRS mutant strains (Figure 9A). Similarly, addition of either *homB* expression vector into the G27Δ*homB* background resulted in biofilm formation that was similar to the other hyper-biofilm forming strains. Of note, the presence of biofilm matrix, flagella, coccoid *H. pylori*, and possible outer membrane vesicles (OMVs) were visible at higher magnification (Supplementary Figure 3). Finally, while both episomal *homB* expression and mutation of the ArsRS system induced aberrant biofilm formation, there did appear to be slight architectural differences between the ArsRS mutant strain biofilms and the biofilms formed when *homB* was expressed *in trans* (Figures 8, 9). Despite these differences, the SEM data suggest that *homB* is intricately involved in the biofilm formation of *H. pylori*.

## Discussion

*H. pylori* persistently colonizes approximately one half of the world's population. In order to persist within the gastric niche, *H. pylori* must adapt to its host and to any changes that occur within that host throughout the course of infection. Given their location, components that make up the cellular envelope, including OMPs, provide key host-pathogen interactions. Comparative studies of sequenced *H. pylori* genomes have identified at least 60 OMPs. Many of these OMPs are among the auxiliary genes that are present in only a subset of strains. HomB, is one of the auxiliary OMPs that is associated with severe disease. Herein, we evaluated *homB* expression patterns, which we reasoned could provide clues about its possible role during *H. pylori* infection.

Initial investigations found that *homB* could be present at two possible loci, and that strains could harbor zero, one, or two copies of *homB* (Oleastro et al., 2008, 2009a, 2010; Kang et al., 2012; Kim et al., 2015). Other OMPs such as the *bab* alleles (*babA, babB*, and *babC*) and the *sab* alleles (*sabA, sabB*, and *hopQ*) also occupy multiple possible loci and can vary in copy number. It is hypothesized that homologous recombination between loci is one way that *H. pylori* regulates OMP expression, which helps it to adapt to its environment (Oleastro et al., 2010). For instance, homologous recombination between *sab* loci has been demonstrated *in vitro* and results in gene duplication (Talarico et al., 2012). Bab genes can also undergo homologous recombination resulting in the formation of chimeric *bab* genes (Backstrom et al., 2004; Solnick et al., 2004). Additionally, a dinucleotide repeat in the 5′ coding region of *bab* locus B leads to phase variation of *bab* alleles present at this locus (Backstrom et al., 2004; Solnick et al., 2004). Translational phase variation is also observed in SabA and SabB (Goodwin et al., 2008). In contrast to the *bab* and *sab* loci, homologus recombination leading to gene duplication or altered regulation has not been demonstrated for *homB*. We found that, the *homB* promoter regions were highly conserved regardless of locus, and unlike many other OMPS, examination of the promoter and coding regions of *homB* revealed no mono/dinucleotide repeats that are typically indicative of slip-strand mispairing or phase variation (Figure 1, Supplementary Figure 1; Salaun et al., 2004). It is worth noting that another member of the Hom protein family, HomA, can also be found at locus A and/or locus B. Prior *in silico* analysis revealed regions of homologous recombination in the middle sections of the *homB* and *homA* genes, which account for the variability among these OMPs (Oleastro et al., 2010); however, this recombination would not account for movement of genes between loci or the conservation of the upstream regions. To our knowledge, such high levels of conservation of the promoter regions of genes found in different loci is not observed for other OMP families. Furthermore, it is unclear if the presence of two *homB* genes versus a single *homB* gene is the result of a single historical duplication event or if this process is fluid during the course of an infection. Our findings suggest future research is needed to elucidate the molecular mechanisms underpinning the genetic rearrangements of *homB* at locus A and locus B. Additionally, we note that while the promoter regions appear identical, the expression of *homB* within a strain that carries *homB* at locus B has yet to be evaluated. Similarly, *homB* expression has not been evaluated in strains carrying *homB* at both loci. Thus, while the data presented here answer some questions about *homB* expression, there are clearly many questions that remain to be answered.

One reason intergenic DNA may be preserved is to maintain the integrity of protein binding sequences that are important for gene regulation. Herein, ArsRS was shown to be a potent regulator of *homB* expression. In fact, in response to low pH, *homB* expression was down-regulated nearly ten-fold in an ArsRS dependent manner. Conversely, despite the fact that *homB* was repressed under conditions of excess nickel, NikR did not appear to directly mediate this change (Figure 3A). Though not completely clear, the nickel responsive change in *homB* expression may in fact be due to changes in regulation of *arsR*; the coordinated regulation of *arsR* expression by Fur and NikR was recently reported (Roncarati et al., 2016). That study found that in the presence of iron, Fur can bind to the *arsR* promoter to repress expression; however, in excess nickel conditions, NikR interferes with Fur repression of *arsR*, which leads to increased ArsR (Roncarati et al., 2016). Thus, it is plausible that the increase in *homB* expression observed in the *nikR* deletion strain is due to the role that NikR plays in ArsR regulation. Also of note, mutations in the ArsRS system not only changed the level of *homB* expression but also changed the pattern of *homB* expression (Figure 2). In the wild-type strain, an increase in *homB* expression was observed during the transition from late-log to stationary phase. An increase in gene expression during the log to stationary transition period is not specific to *homB* and this transition in gene expression has been coined as the “log-stat switch” (Thompson et al., 2003). In fact, numerous virulence factors are among the genes shown to increase during the growth phase transition, suggesting that perhaps *homB* is up-regulated as part of *H. pylori*'s virulence arsenal (Thompson et al., 2003). In the ArsRS mutant strains a dramatic increase in expression was still observed; however, it occurred during log-phase (Figure 2B). These data suggest that while ArsRS was responsible for suppression of *homB* expression, another regulatory factor may be involved in the increase in *homB* expression that was observed at T48 and T20 in the wild-type and Δ*arsS* strains, respectively. It is plausible that expression of *homB* is regulated both positively and negatively by multiple environmental cues, which opens the opportunity for future investigations. In addition, we observed subtle differences between the ArsR-D52N mutant strains and the Δ*arsS* strain. On average, a higher level of *homB* expression was observed in the ArsRS-D52N mutant strain as compared to Δ*arsS* strain (Figures 2C, 3B). One explanation for this differences in *homB* expression could be that a low level of ArsR~P actually remains in the Δ*arsS* strain; ArsR~P could be generated via spontaneous or nonspecific phosphorylation and could then bind to the *homB* promoter. Conversely, the ArsR-D52N strain would never be able to be phosphorylated spontaneously or nonspecifically; the crucial residue is mutated. Given this possibility, we note that herein we have assessed binding of ArsR~P to the *homB* promoter. However, future studies should examine the structure function relationships between this interaction and should examine the ability of purified ArsR-D52N to bind the *homB* promoter using a technique such as fluorescence anisotropy. Such studies will allow us to address difference in the binding kinetics between the different forms of ArsR (ArsR, ArsR~P, and ArsR-D52N) and could provide valuable information on the exact DNA binding site needed for binding and regulation.

In a recent publication we proposed a model in which ArsRS senses the pH at the gastric epithelium and modulates gene expression to promote colonization and biofilm formation (Servetas et al., 2016). Given the location of HomB in *H. pylori*'s outer membrane, and the increase in *homB* expression observed following ArsRS mutation, we reasoned that HomB may contribute to biofilm formation. In fact, at 24 h, a significant increase in *homB* expression was observed in the biofilm associated bacteria as compared to the planktonic cells (Figure 6). Furthermore, *homB* was not only integral to the hyper-biofilm phenotype observed in the ArsRS mutant strains (Figures 5, 8), but expression of *homB in trans* also enhanced biofilm formation in wild-type and the Δ*homB* strain backgrounds (Figures 7, 8). Surprisingly, even the subtle increase in *homB* expression in the G27Δ*homB* pFlaA*-homB* strain (Figure 7), appeared to be sufficient to induce biofilm formation (Figure 9, Supplementary Figure 3). While the level of *homB* expression was not always dramatically increased, the pattern of *homB* expression over time was changed in all of the hyper-biofilm strains. In wild-type *H. pylori, homB* expression didn't change dramatically until 48 h (Figure 2A) whereas in the Δ*arsS* strain, the ArsR-D52N strain, or in strains where *homB* was expressed *in trans, homB* expression was altered (Figures 2, 6). These data suggest that over expression of *homB* as well as aberrant timing of *homB* expression may both contribute to the hyper-biofilm phenotype.

While our data indicate that HomB is a crucial component of the *H. pylori* biofilm, numerous questions about biofilm formation remain. For example, do other OMPs contribute to biofilm development? This question is of particular interest given that higher levels of biofilm formation were observed in the Δ*arsS* strain as compared to the ArsR-D52N strain and given that deletion of *homB* didn't completely ablate biofilm formation. While the differences in biofilm between the ArsRS mutant strains may be related to *homB*, ArsRS regulates numerous genes, including other OMPs. Clearly, biofilm formation is a multifactor processes and it is highly likely that multiple genes that contribute to biofilm formation are affected, either directly or indirectly, by the ArsRS two component system. Thus the difference in biofilm formation between the two ArsRS mutant strains may be related to the compounding of subtle differences in gene expression between the two strains.

To date, a few other OMPs have been referenced in terms of biofilm formation (Wong et al., 2016; Yonezawa et al., 2017). A high-throughput screen identified the presence of another gene within the Hom family, *homD*, in association with *H. pylori* strains that formed robust biofilms; however, no molecular work has been done to verify the role of this OMP in biofilm development (Wong et al., 2016). Conversely, the OMP, AlpB was evaluated more fully and appears to play a considerable role in biofilm formation in *H. pylori* strain TK1402 (Yonezawa et al., 2017). Furthermore, mutation of a single domain in AlpB, which is predicted to be in an environmentally exposed loop, effected biofilm development by TK1402 (Yonezawa et al., 2017). Notably, differences between the HomB and HomA paralogs are also predicted to reside in environmentally exposed loops (Servetas et al., 2018). Thus, these findings present an interesting avenue to pursue in the identification of functional difference between HomA and HomB. It is also of note that several other OMPs have been identified within the ArsRS regulon; *sabA, labA*, and *hopZ* were all recently shown to be subject to ArsRS-dependent regulation by Acio-Pizzarello et al. (2017). Similar to *homB*, strains lacking ArsS show an increase in expression of *sabA, labA*, and *hopZ* (Acio-Pizzarello et al., 2017). Thus, it is plausible that these other ArsRS regulated OMPs may contribute to the biofilm formation that remains in the Δ*homB* strains (Figures 5, 7, 8).

In summary, the data presented herein demonstrated that aberrant *homB* expression was necessary and sufficient to induce hyper-biofilm formation by *H. pylori* G27. Given the heterogeneity of *H. pylori* OMP profiles seen across *H. pylori* strains and the differences in the propensity for biofilm formation seen between *H. pylori* strains, the work presented here highlights areas for future investigation; what are the roles of other OMPs in biofilm development, and what consequences do variable OMP genotypes play on biofilm formation in *H. pylori*?

## Author contributions

SS provided the experimental design, conducted experiments, preparation of manuscript. DM provided the experimental design, preparation of manuscript. AK, J-HC sequenced *homB* locus A and locus B promoter regions from clinical isolates. RD, JG conducted the SEM studies. IW aided in biofilm experiments.

### Conflict of interest statement

The authors declare that the research was conducted in the absence of any commercial or financial relationships that could be construed as a potential conflict of interest.
