# Supplementary material for: ArsRS-Dependent Regulation of homB Contributes to Helicobacter pylori Biofilm Formation
Source: Front Microbiol. 2018 Aug 2;9:1497. doi: 10.3389/fmicb.2018.01497 (PMC6083042; doi:10.3389/fmicb.2018.01497)
Supplement: Supplementary file 1 [file Image_1.PDF]

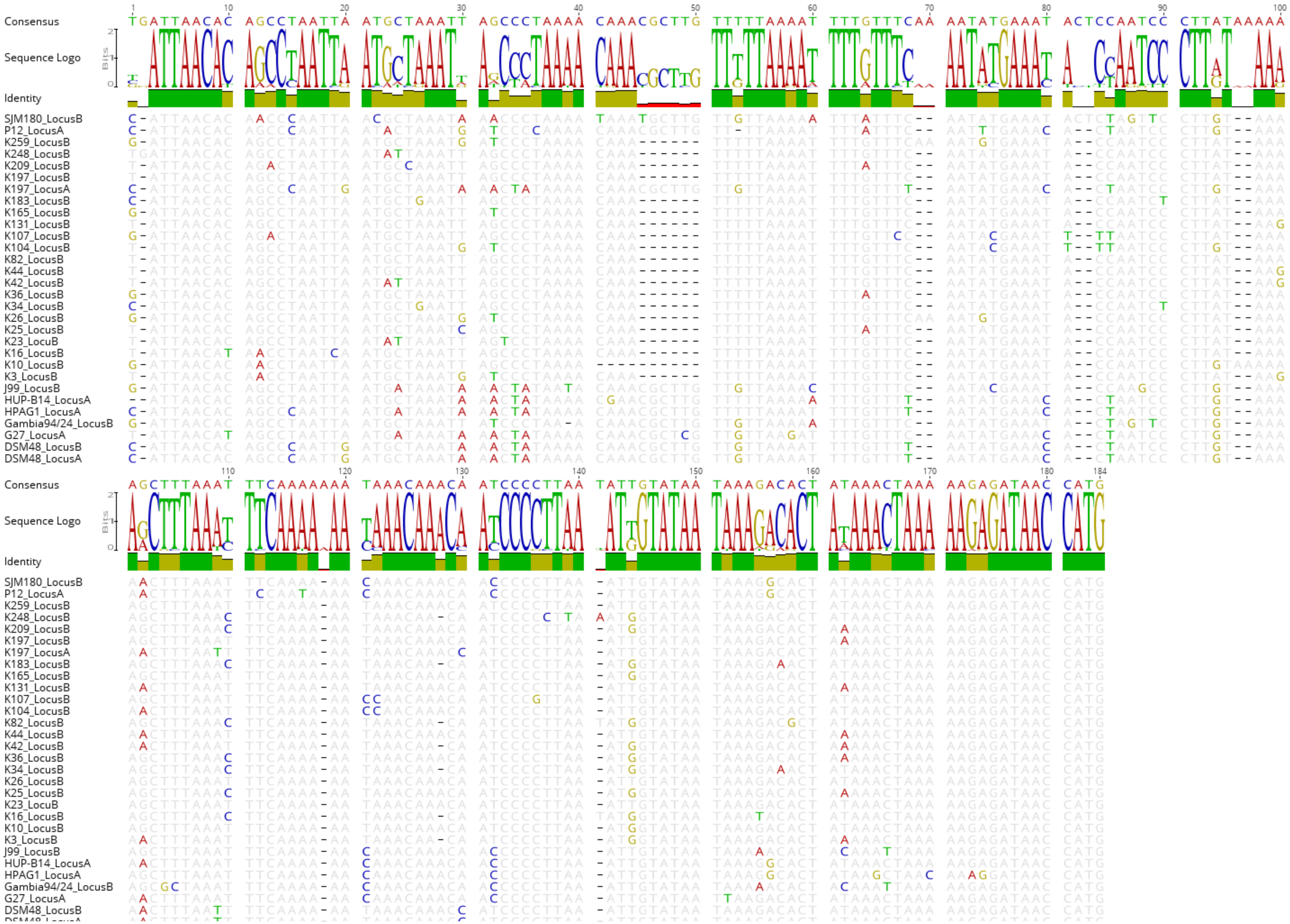
Consensus
Sequence Logo
Identity
Bits
TGATTAACAC AGCCTAATTA ATGCTAAATT AGCCCTAAAA CAAACGCTTG TTTTTAAAAT TTTGTTTCAA AATATGAAAT ACTCCAATCC CTTATAAAAA
AGCTTTAAAT TTCAAAAAAA TAAACAAACA ATCCCCTTAA TATTGTATAA TAAAGACACT ATAAACTAAA AAGAGATAAC CATG
SJM180_LocusB
P12_LocusA
K259_LocusB
K248_LocusB
K209_LocusB
K197_LocusB
K197_LocusA
K183_LocusB
K165_LocusB
K131_LocusB
K107_LocusB
K104_LocusB
K82_LocusB
K44_LocusB
K42_LocusB
K36_LocusB
K34_LocusB
K26_LocusB
K25_LocusB
K23_LocuB
K16_LocusB
K10_LocusB
K3_LocusB
J99_LocusB
HUP-B14_LocusA
HPAG1_LocusA
Gambia94/24_LocusB
G27_LocusA
DSM48_LocusB
DSM48_LocusA

Supplemental Figure 1. Alignment of *homB* promoter regions

184 nucleotides upstream of the *homB* translation start site were aligned from 30 *homB* loci from 29 strains. The name to the left of each sequence denotes the strain and the locus. At the top of the alignment, a consensus sequence is given. Below the consensus sequence is a WebLogo that shows the conservation across the 30 loci; the larger the letter, the more highly conserved the corresponding base pair. To indicate nucleotides where substitutions/deletions are present, the letter(s) at these positions do not reach the full height. Within the alignment, nucleotides in grey indicate conservation at all 30 loci, colored nucleotides indicate the presence of SNPs, and dashes designate gaps.
