# Supplementary material for: ArsRS-Dependent Regulation of homB Contributes to Helicobacter pylori Biofilm Formation
Source: Front Microbiol. 2018 Aug 2;9:1497. doi: 10.3389/fmicb.2018.01497 (PMC6083042; doi:10.3389/fmicb.2018.01497)
Supplement: Supplementary file 2 [file Image_2.PDF]

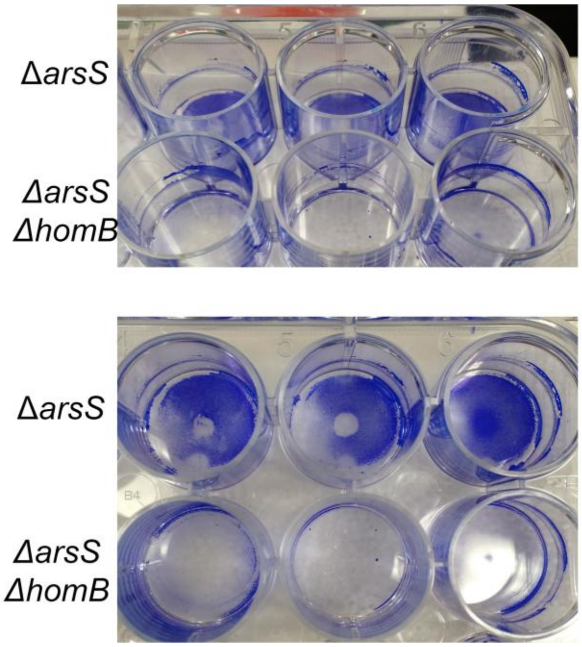

Supplemental Figure 2. Crystal Violet staining of Δ*arsS* and Δ*arsS*Δ*homB* biofilms
Representative images show 48 hour Δ*arsS* and Δ*arsS*Δ*homB* biofilms stained with crystal violet. The same biofilms are shown in both pictures from an oblique (top) and overhead (bottom) angle. Each strain was grown and is shown in triplicate. Biofilm formation at the air-liquid interface can be observed in both strain; however prominent staining on the bottom of the well is absent in the Δ*arsS*Δ*homB* strain.
