# Supplementary material for: ArsRS-Dependent Regulation of homB Contributes to Helicobacter pylori Biofilm Formation
Source: Front Microbiol. 2018 Aug 2;9:1497. doi: 10.3389/fmicb.2018.01497 (PMC6083042; doi:10.3389/fmicb.2018.01497)
Supplement: Supplementary file 3 [file Image_3.PDF]

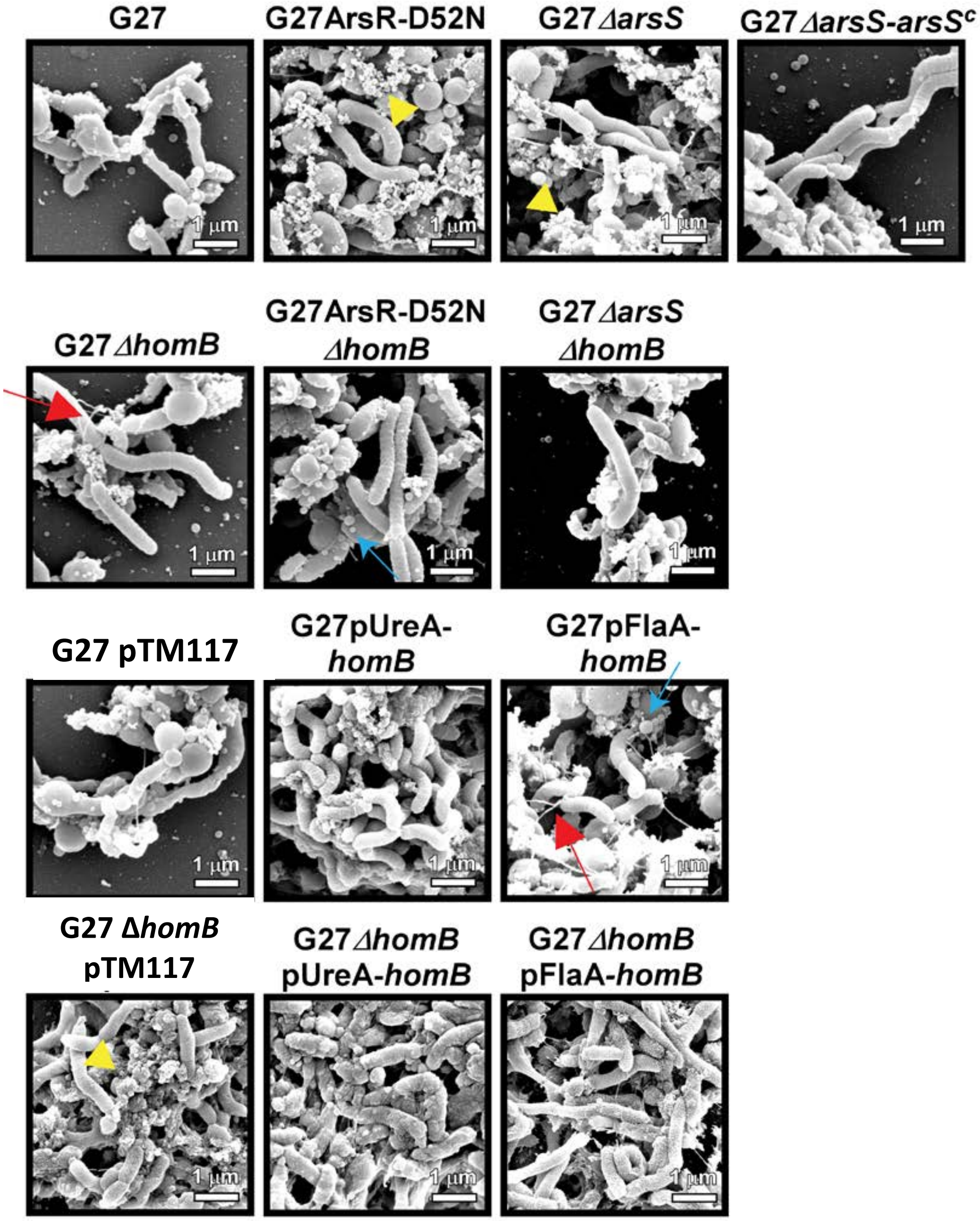

Supplemental Figure 3. SEM of *H. pylori* biofilms at 20,000X magnification
Representative SEM images of 48 hour biofilms are shown at 20,000X magnification. Across the images, prototypical spiral shaped *H. pylori* can be observed. Additionally, presumptive structures, such as flagella (red arrow), OMVs (blue arrow), and biofilm matrix (yellow arrowhead), can also be seen.
